# A Comparative Study on Growth Performance, Body Composition, and Liver Tissue Metabolism Rearing on Soybean Lecithin-Enriched *Artemia* Nauplii and Microdiet in Rock Bream (*Oplegnathus fasciatus*) Larvae

**DOI:** 10.1155/2023/5545898

**Published:** 2023-03-16

**Authors:** Pian Zhang, Peng Tan, Lei Zhang, Wenliang Zhu, Ruiyi Chen, Ligai Wang, Dongdong Xu

**Affiliations:** ^1^Fisheries College, Zhejiang Ocean University, Zhoushan 316022, China; ^2^Key Laboratory of Mariculture and Enhancement, Zhejiang Marine Fisheries Research Institute, Zhoushan 316021, China; ^3^Marine and Fisheries Research Institute, Zhejiang Ocean University, Zhoushan 316022, China

## Abstract

This study is aimed at establishing optimal soybean lecithin (SL) enrichment protocols in *Artemia* nauplii and at comparing the growth performance, body composition, and liver tissue metabolism in rock bream (*Oplegnathus fasciatus*) larvae reared on SL-enriched *Artemia* nauplii or SL-enriched microdiet (MD). The enrichment protocol experiment results indicated 12 h enrichment, and 10 g SL/m^3^ seawater could obtain desirable results. Rock bream larvae (25 days posthatching (dph)) were fed *Artemia* nauplii or MD for 30 days with three replicates. At stage 1 (larval 25–40 dph), significantly higher growth performance was observed in larvae fed the live prey (*P* < 0.05). Conversely, at stage 2 (41–55 dph), feeding with MD significantly increased larval standard length, and specific growth rate compared with those of larvae fed live prey. Larvae fed a MD showed decreased lipolysis-related lipase activity as well as decreased amino acid catabolism-related alanine aminotransferase and aspartate aminotransferase enzyme activities in liver tissue. RNA sequencing revealed that feeding with the MD primarily increased the expression of lipogenesis-related genes and protein translation-related gene expression in the liver tissue. Notably, feeding with MD significantly increased ribosome biogenesis-related genes as well as mitochondria synthesis-related gene expression, indicating a high protein anabolism rate and high energy production in liver tissue. In conclusion, 10 g SL/m^3^ seawater and 12 h could effectively enrich SL in *Artemia* nauplii. Retard weaning onto MD led to lower growth performance, which was likely due to the diversity of lipid and protein metabolism.

## 1. Introduction

Successful larval rearing is a bottleneck in marine fish culture. In this regard, addressing larval fish nutrition is complex but essential to obtain high-quality and large-quantity fingerlings. Primarily, there are two ways to approach larvae in captivity: the first is rearing on live prey such as rotifers and brine shrimp (*Artemia* sp.), and the second is feeding a microdiet (MD). Most species cultured to date require the use of high digestive live prey, primarily because of the incapability of developing a complete, functional, fully developed digestive system at very early life stages [1]. Considering that live prey is difficult to sustain, requires considerable space and expense, and is certainly nutrient deficient, the substitution of live prey as early as possible with MD is the main obstacle to larval nutrition [2]. It is well recognized that MD is easier to maintain and has lower production costs; however, weaning at the early larval stage could result in progressive starvation, retarded growth performance, and high mortality [3–5]. These undesirable results cannot be attributed solely to the unbalanced nutritional quality of the diets; other factors have been conjectured to explain the lower performance of MD, such as low ingestion rates. In this sense, the application of nutrition-balanced food sources (live prey or MD) should be carefully considered to establish an optimal rearing strategy during fish larval stages [6, 7].

Among the different sources of live prey, *Artemia* is the most frequently used in cultured fish and crustaceans. However, their suitability as a diet for marine larvae has often been questioned because *Artemia* may lack certain nutrients pertinent to their target species, such as DHA, EPA, and phospholipids (PL) [8, 9]. Marine fish larvae have been proven to have no or negligible capacity in de novo biosynthesis of phospholipids; therefore, balanced nutrition should be designed to ensure the provision of an extra level of PLs for fish early life stages [10]. *Artemia* nauplii passively filter enriched materials, and their digestive tract becomes loaded with any particle of suitable size present in the medium [11]. Therefore, *Artemia* nauplii can become vehicles of enrichment products, and the enrichment outcomes can be manipulated depending on the targeted organisms. A study reported the beneficial roles of PL enrichment in *Artemia franciscana* nauplii in promoting growth at the live prey feeding stage in green terror cichlid (*Aequidens rivulatus*) larvae [12]. Likewise, our recent study found that SL-enriched *Artemia* nauplii (12 h, 10 g/m^3^ seawater) significantly improved the growth performance of yellow drum larvae (12–22 dph) compared with the growth performance of larvae fed newly hatched *Artemia* (data unpublished). Studies have proposed that the beneficial effects of SL could be attributed to its dietary emulsifying properties [13], lipid absorption [14], transportation promotion [15], and digestive enzyme activities [2, 16]. Mounting evidence has shown the beneficial effects of PL supplementation in MD at the larval stage; nevertheless, only a few studies have focused on the SL enrichment protocol in *Artemia* nauplii. A previous study noted that *Artemia* metanauplii could be successfully enriched with PLs in a short-term incubation (4 h) [17]. In contrast, *Artemia franciscana* nauplii were reported to be enriched with SL for 24 h at 0.6 g/m^3^ seawater [12]. Enrichment can improve *Artemia's* nutritional profiles, while enrichment procedures could lead to undesired effects such as decreased nutrition values. Despite the importance of PL enrichment in *Artemia*, there remains a paucity of evidence on protocols. Establishing optimized protocols for SL in *Artemia* is a challenge that needs to be urgently addressed.

One of the most important species for enhancement in the East China Sea is the rock bream (*Oplegnathus fasciatus*). To date, the rock bream larva rearing strategy is still defective. Many studies have centered on evaluating larval nutritional conditions by assessing the rate increase in size, length, or biomass and morphological features, as well as determining the activity of different metabolic enzymes by means of biochemical quantification. Recently, those approaches have been complemented by molecular biological techniques that provide insight into genes involved in the development, metabolism, and even immunity. Molecular biology research technologies such as a unique molecular identifier (UMI) mRNA duplicates can now be accurately classified thanks to RNA sequencing [18]. This approach offers a useful tool for investigating mechanisms relating to early larval feeding sources. The primary tissue for nutrient digestion and absorption is the gut. For food digestion, absorption, and subsequent nutrient acquisition, a well-developed intestinal structure is crucial. In a recent study, we found that intestinal morphology development in larvae of yellow drum (*Nibea albiflora*) and this species was influenced by the high inclusion level of dietary SL [19, 20]. The liver is the central digestive organ that plays a significant part in the anabolism, catabolism, and reallocate of nutrients, primarily proteins and lipids. Further studies are warranted to investigate how PL inclusion influences liver metabolism. The ultimate goal of this study was (1) to identify the most effective *Artemia* nauplii enrichment strategy with respect to SL dosage and time in order to increase larval quality and survival and (2) to compare the growth performance, body composition, and liver tissue metabolism in rock bream larvae reared on SL-enriched *Artemia* nauplii or MD.

## 2. Materials and Methods

According to the Guide for the Care and Use of Laboratory Animals formulated by the Ministry of Science and Technology of China, all experimental larvae used in the present study were carefully handled. The study was approved by the Committee on Ethics of Animal Experiments of Zhejiang Ocean University and was performed in compliance with the ethical principles and standards of Aquaculture Nutrition. Prior to sampling, the larvae were anesthetized with 10 mg L^−1^ tricaine methanesulfonate (MS-222, Sigma, USA).

### 2.1. Chemical Analysis

According to the methods described by the Association of Official Analytical Chemists [21], the crude protein, crude lipid, moisture, and ash contents of the raw ingredients, larval body, live prey, and experimental diet were determined with two replicates for each sample. The sample was placed in an oven at 105°C for 7 h to a constant weight to obtain the moisture content. The Kjeldahl method with an automatic digester (KjelFlex K-360, BUCHI, Switzerland) was adopted to analyze the sample crude protein content. Crude lipid extraction and determination were conducted using a Soxtec System HT (Soxtec 2055, FOSS Tecator, Sweden). A muffle furnace at 550°C for 6 h was applied to burn off the organic matter to obtain the sample ash content.

Total lipid isolation and purification procedures were in accordance with a previous description [22]. The procedures for fatty acid methyl ester (FAME) preparation were described previously [23]. Fatty acids were finally presented as mol % of total identified fatty acids detected. The amino acid content was described according to our previous publication and expressed as % dry weight [24]. Determination of PL content in live prey was conducted using the molybdenum blue colorimetry method as described in our previous publication [19].

### 2.2. Soybean Lecithin Enrichment in *Artemia* Nauplii

According to our previous description, soybean lecithin (SL, Cargill, Germany) was gently dissolved in boiling water [19]. Newly hatched eggshells of *Artemia* nauplii (~2 × 10^5^/L) were transferred into the enrichment tank. *Artemia* nauplii were enriched at a temperature of ~28°C with SL for 0 h, 3 h, 6 h, 12 h, and 24 h with gradient dosages of 0 g/m^3^, 5 g/m^3^, 10 g/m^3^, and 20 g/m^3^ SL, respectively. Each treatment was conducted with three replicates. For live prey sample collection, samples were centrifuged to remove seawater, and the live prey was collected and stored at −20°C until further analysis or stored at 4°C before feeding.

### 2.3. Microdiet Preparation

The majority of the MD formulation was based on our earlier research by Tan et al. [19] (Table S1). The feed preparation method refers to our previous study [20], and all feed ingredients are ground into fine powder, then mixed well with liquid ingredients, then pelletized on the machine, and finally ripened. Three distinct particle sizes of the diets were offered: above 178 *μ*m, 125–178 *μ*m, and below 125 *μ*m, respectively. Before feeding trials, the prepared MDs were packaged and kept at −20°C.

### 2.4. Larval Rearing

The larvae were first reared with rotifers (*Brachionus rotundiformis*) (3–20 dph) and then coreared with rotifers and *Artemia* nauplii (21–22 dph). Before the larval feeding experiment, 1200 larvae were randomly divided into 6 tanks with 200 larvae per tank at larval 22 dph. Larvae were reared in a flowing water system with a water flow rate of ~0.5 L/min. The water temperature is 21–24°C. The salinity of the culture water is 27 ± 1*‰* and dissolved oxygen > 6.0 mg/L. Larvae were cofed with MD and *Artemia* nauplii for 22–24 dph prior to the MD treatment to acclimate the larvae. During this period, to achieve larval qualities that stayed constant, deceased larvae were drained away and new ones were added. Larvae with an initial body weight of 51.0 mg and an initial body length of 1.01 cm were selected. Larvae were given the MD every two hours from 6 : 00 to 20 : 00 during the feeding trial. To ensure larvae were fed with satiation, we keep a small amount of MD at bottom of the tank until the beginning of the next feeding. The tank was cleaned twice daily by siphoning off waste, remaining bait, and larval corpses. During the cleaning process, the bodies were meticulously identified and recorded twice a day. MD particles less than 125 *μ*m in size were supplied to the larvae at a rate of 25–34 dph as part of the treatment. Afterward, the larvae were fed with larger particle-sized MD. For the art group, larvae were fed with *Artemia* nauplii enriched with SL for 12 h and 10 g/m^3^ seawater.

### 2.5. Sample Collection

Before the start of the feeding trial, larval initial body weight (BW) and standard length were obtained by averaging the total weight and total standard length of 50 larvae. Larvae from each tank were randomly selected at the conclusion of feeding stage 1 (larvae 40 dph) to be measured for BW and standard length. After preserving 20 larvae per tank for air exposure challenge, the remaining larvae were anesthetized and sacrificed to obtain the standard length and BW. The liver tissue was randomly selected from ten larvae each tank. These liver tissue samples were immediately frozen in liquid nitrogen and stored at −80°C until enzyme activity analysis. Three larvae per tank were anesthetized and sacrificed to obtain liver tissue, until complete RNA extraction, and tissues were kept in RNA Later (Thermo Fisher Scientific, USA) at −20°C. The remaining larval corpses were collected in tanks and kept at −20°C until their proximate composition and fatty acid and amino acid profiles were analyzed.

### 2.6. Hypoxia Stress Challenge

The main tests were carried out to establish the ideal period for the hypoxic stress challenge. Following the sampling, the remaining larvae were caught in a mesh net and exposed to air for precisely 300 seconds. The larvae were immediately put back in their own aquarium after that. The dead fish were gathered during the course of the following 24 hours, and the quantity was noted.

### 2.7. Enzyme Activity Analysis

Sample preparation was conducted as in a previous publication [19]. According to the manufacturer's instructions, commercial kits (total protein quantitative assay kit, alanine aminotransferase (ALT) kit, aspartate aminotransferase (AST) kit, and lipase (LP) assay kit, Nanjing Jiancheng Bioengineering Institute, China) were used to ascertain the total protein content and ALT, AST, and LP enzyme activities. A visible spectrophotometer was used to conduct a colorimetric analysis (Biochrom Ultrospec 2100 pro, England).

### 2.8. UMI-RNA-Seq and Bioinformatics Analysis

The total RNA of the liver tissue from larvae fed live prey and MD was extracted using TRIzol reagent (Invitrogen, USA). RNA integrity, purity, and concentration were determined according to our previous description [25]. RNA sequencing was performed with the assistance of the Experimental Department of Novogene Co., Ltd. (Beijing, China). The construction of RNA-seq libraries, quality evaluation and assembly, annotation, and differentially expressed gene (DEG) analysis refers to our previous study by Tan et al. [19].

### 2.9. DEG Validation by Real-Time Quantitative PCR

Total RNA was extracted from the identical sample used for Illumina sequencing. Reversed cDNA was synthesized using a PrimeScript RT reagent kit with a gDNA eraser (Takara, Japan) following the manufacturer's instructions. *β*-Actin was used as the internal control for the normalization of gene expression levels. The gene expression profiles of the 14 target genes were validated by real-time quantitative PCR (qRT–PCR). The primers used are summarized in Table S2. PCR was performed using an ABI real-time PCR machine (ABI Step One Plus, ABI, USA). The program settings used were the same as those described in our previous study [24]. The comparative CT approach was used to determine expression levels [26].

### 2.10. Calculations and Data Statistics

The following variables were calculated:

(1) $$\begin{array}{c} \text{Final body weight}\left(\text{FBW},\text{g}\right)=\frac{W_{t}}{N_{t}}, \end{array}$$

(2) $$\begin{array}{c} \text{Survival rate}\left(\text{SR},\%\right)=100\times \frac{N_{t}}{N_{0}}, \end{array}$$

(3) $$\begin{array}{c} \text{Survival rate after hypoxia stress }\left(\%\right)=100\times \frac{\text{larvae survival quantity}}{\text{number of larvae for the stress test}}, \end{array}$$

(4) $$\begin{array}{c} \text{Specific growth rate }\left(\text{SGR},\%/\text{d}\right)=100\times \frac{\left[\text{Ln}\left(W_{t}/N_{t}\right)-\text{Ln}\left(W_{0}/N_{0}\right)\right]}{t}, \end{array}$$

(5) $$\begin{array}{c} \text{Weight gain rate }\left(\text{WGR},\%\right)=100\times \frac{\left(W_{t}-W_{0}\right)}{W0}, \end{array}$$

where *W*_*t*_ and *W*_0_ are the sums of the final and initial larvae BWs, respectively. In each tank, *N*_*t*_ represents the end larval population, *N*_0_ represents the initial larval population, and *t* represents the experiment's length in days. GraphPad Version 9.0.0 for Windows (USA, http://www.graphpad.com) was used to process all the data and shown as the mean ± standard error of the mean (S.E.M.). The Levene test was used to verify the data's normality and homogeneity of variances. The effects of SL dosage (factor 1), enrichment time (factor 2), and their interactions on approximate nutrient composition, fatty acid composition, and amino acid composition were analyzed by two-way ANOVA. If the effects of independent factors were significant; then, these effects were evaluated separately by one-way ANOVA and Duncan's multiple range as post hoc tests were performed. *P* < 0.05 was considered significant for all statistical tests. String (http://string-db.org) was used to make the DEG association network prediction before being further processed with Cytoscape software (http://www.cytoscape.org). [27]. We generated the multidimensional correlation analysis using the ggplot2, linkET, and dplyr R packages.

## 3. Results

### 3.1. *Artemia* Nauplii Proximate Nutrients, Fatty Acids, and Amino Acid Analysis

According to proximate nutrient composition analysis (Table 1), *Artemia* nauplii PLs were enriched with increased SL dosage (dosage, *P* < 0.001) and enrichment time (time, *P* < 0.001) in an interactive mode (*D* × *T*, *P* < 0.001). The medium SL dosage showed no significant effect on *Artemia* protein, moisture, and ash content (dosage, *P* > 0.05). SL enrichment significantly increased the *Artemia* nauplii lipid content in a dosage-dependent manner (Table 1, dosage, *P* < 0.001).

No significant differences in *Artemia* nauplii fatty acid composition were observed, regardless of SL dosage (Table 2, dosage, *P* > 0.05). Live prey enrichment time significantly changed the SFA, MUFA, and n-3 LC-PUFA proportions (Table 2, *P* < 0.05). Amino acid content analysis indicated that live prey enrichment time was the primary factor that affected the content of the amino acids (time, *P* < 0.001) (Table 3).

### 3.2. Food Sources on Larval Growth Performance

**Figure 1:**
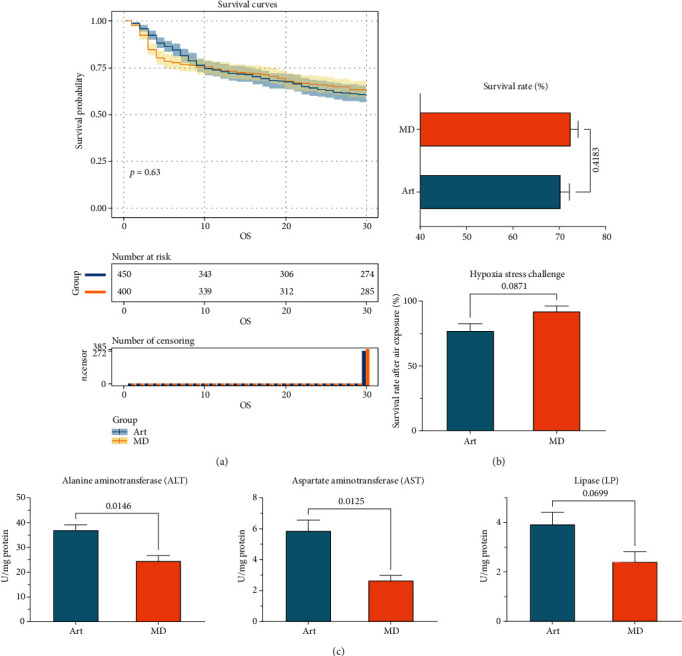
Rock bream larval survival rate, hypoxia stress resistance, and liver tissue metabolism-related enzyme activities. (a) Daily data on the rock bream larval survival rate were collected during the feeding study. The right panel displays the results of the statistical analysis. (b) Larval stress tolerance after 300 seconds of air exposure. (c) Larval liver tissue amino acid metabolism-related and lipid metabolism-related enzyme activity corresponds to *Artemia* and MD.

At the stage 1 (larvae 40 dph), no significant difference in standard length and SGR was observed between larvae fed with the live prey and MD (*P* > 0.05). Significantly higher BW (*P* < 0.05) and WGR (*P* < 0.05) were observed in larvae fed the live prey than in those fed the MD (Table 4). At the end of stage 2 (larvae 41–55 dph), significantly higher growth performance was observed in larvae fed MD, characterized by higher standard length (*P* < 0.01), WGR (*P* < 0.001), and SGR (*P* < 0.001). Interestingly, the larval retarded growth performance at 25–40 dph resulting from feeding with MD was completely reversed by feeding with MD, reaching a comparable BW at larvae 55 dph (*P* > 0.05). The siphon method was used to gather the bodies, and the number of bodies was meticulously recorded twice daily. During the experiment, the number of corpses was carefully recorded daily, and survival curves were plotted (Figure 1(a)). At the end of the feeding trial, statistical results indicated no significant difference in SR (Figure 1(a), *P* = 0.4183) or hypoxia stress challenge survival rate (Figure 1(b), *P* = 0.0871) between groups.

### 3.3. Larval Body Proximate Nutrient, Fatty Acid, and Amino Acid Profiles

Table 5 compares larval body proximate nutrients, fatty acids, and amino acids profiles of larvae reared on different food sources (larval 55 dph). Significantly lower crude lipid content (*P* < 0.001) but significantly higher ash content (*P* < 0.05) and moisture content (*P* < 0.05) was observed in larvae fed the MD compared with those of larvae fed the live prey. Larvae fed the MD showed significantly higher SFA (*P* < 0.001), n-3 PUFA (*P* < 0.01), n-6 PUFA (*P* < 0.05), and n-3 LC-PUFA (*P* < 0.01) proportions but significantly lower MUFA (*P* < 0.001) proportions than larvae fed live prey. Feeding with the MD, rather than feeding with the live prey, significantly increased the larval body EAA, NEAA, and TAA content (*P* < 0.05). Correlation analysis indicated that live prey fatty acid composition (*R*^2^ = 0.61, *P* < 0.001), amino acid content (*R*^2^ = 1.00, *P* < 0.001), and proximate nutrient composition (*R*^2^ = 0.81, *P* < 0.001) were all highly correlated with those of larvae fed live prey. The fatty acid composition (*R*^2^ = 0.62, *P* < 0.001) and amino acid content (*R*^2^ = 1.00, *P* < 0.001) were highly correlated between the MD and larvae fed the MD. The proximate nutrient composition was not significantly correlated between the MD and larvae fed the MD (*R*^2^ = 0.07, *P* > 0.05).

### 3.4. Larval Liver Tissue Metabolism-Related Enzyme Activities

Liver tissue metabolism-related enzyme activity results indicated that feeding with MD decreased lipase (LP) (*P* = 0.0699), aspartate aminotransferase activity (AST) (*P* = 0.0.0125), and alanine aminotransferase (ALT) (*P* = 0.0146) (Figure 1(c)).

### 3.5. Liver Tissue RNA-Seq Quality Control, Mapping, and Bioinformatics Analysis

**Figure 2:**
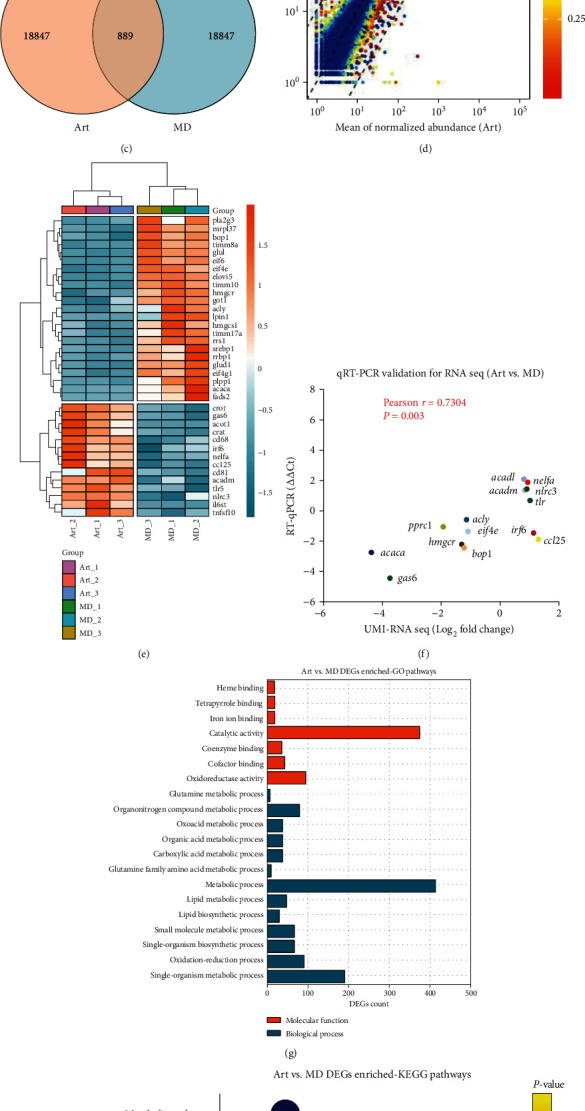
Transcriptome analysis of larval liver tissue. (a) Pearson's correlation between samples. (b) PCA of samples. (c) Number of DEGs presented in a Venn diagram. (d) Volcanic map of detected gene expression levels between the art and MD groups. (e) Heat map presentation for partial DEGs between treatments. (f) UMI-RNA-seq DEG validation by qRT–PCR. (g, h) DEGs enriched GO and KEGG pathways between groups.

Detailed information on the sequencing data and mapping is summarized in Table S3. Clean reads were mapped on the annotated genome of rock bream, and an average of 96.88% of the UMI reads could be successfully mapped, of which an average of 89.42% was uniquely mapped. Pearson correction analysis between samples indicated high correction between treatments (Figure 2(a)). The read-count approach identified 889 significant DEGs in the art versus MD groups, of which 366 were strongly upregulated and 523 were considerably downregulated. (Figures 2(b) and 2(c)). Some protein metabolism-related genes, such as *eif4e* and *eif4g1*, in addition to lipid metabolism-related genes, such as *hmgcr*, *acaca*, *elovl5*, and *srebp1*, were observed among the DEGs. Notably, mitochondrial synthesis-related genes (*timm10*, *timm8a*, *timm17a*, and *pprc1*) and ribosome synthesis-related genes (*mrpl37*, *rrbp1*, *bop1*, and *rrs1*) were significantly differentially expressed between the groups. Specially chosen DEGs that were involved in metabolism were displayed in the heat map (Figure 2(d)). Gene expression levels were distinguished by the variety of block colors (Figure 2(e)).

In order to verify the DEGs discovered by RNA-seq analysis, we carried out qRT-PCR on 14 chosen genes (Figure 2(f)). According to the qRT-PCR study, all genes had similar expression patterns to those found in the transcriptome data (*ΔΔ*Ct versus Log_2_-fold change). According to a Pearson correction analysis, the correlation between these two approaches was 0.7304, with a *P* value 0.003. The validity of the UMI-RNA-seq data used in this investigation was further supported by the qRT-PCR results.

Gene Ontology (GO) analysis was performed on all DEGs, and the results revealed that DEGs were significantly enriched in the biological processes and molecular function categories. The most enriched GO pathways among them were those related to metabolic processes and single-organism metabolism (Figure 2(g)).  Figure 2(h) displays the top 20 KEGG (Kyoto Encyclopedia of Genes and Genomes) pathways. The metabolic pathway was the KEGG pathway with the highest rich factor, with the involvement of pathways related to protein and lipid metabolism (blue and purplish-red characters, respectively), as well as several other pathways (black character).

**Figure 3:**
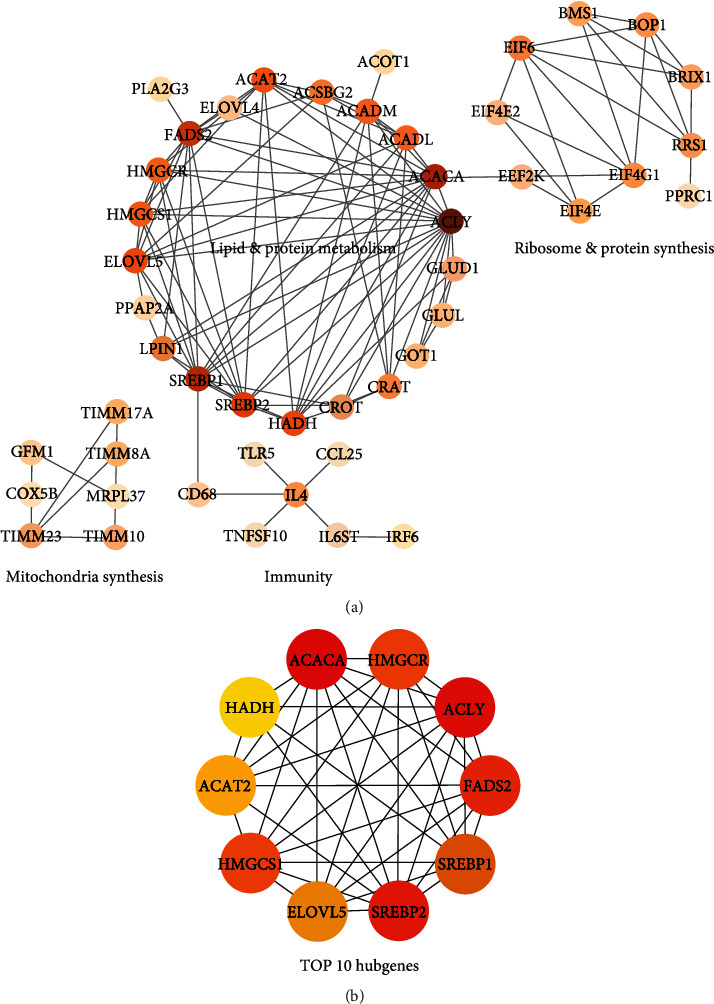
Potential association network for DEGs predicted by Cytoscape software with high confidence. (a) DEGs were clustered into 4 categories, namely, lipid and protein metabolism, mitochondria synthesis, immunity, and ribosome and protein synthesis. (b) Top 10 hub genes calculated by the cytoHubba plugin.

String was used to predict the DEG association network with high confidence, and Cytoscape was used to build the DEG interaction network.  Figure 3(a) displays a cluster demonstrating several gene interactions linked to lipid and protein metabolism, including those between the genes *acaca and acly*, *hmgcr*, and *srebp1*, as well as some interactions related to protein metabolism, including those between the genes *glud1*, *got*, *and glul*. The cluster demonstrating gene connections related to mitochondrial biosynthesis is the main focus of the bottom left panel. The genes for the essential proteins *timm7*, *timm8a*, *timm23*, and *cox5b* are essential in mitochondria synthesis. The bottom middle panel clustered some immune-related genes, such as *cd68*, *tlr5*, *ccl25*, and *il4*. The upright panel displays a cluster demonstrating the translation of important proteins for ribosome biogenesis, maturation, and assembly by the genes *eif4e*, *eif4g1*, *eif4e2*, *bop1*, and *rrs1*, as well as mRNA translation and protein synthesis.  Figure 3(b) highlights the top 10 hub genes, namely, *acaca*, *hmgcr*, *acly*, *fads2*, *srebp1*, *srebp2*, *elov5*, *hmgcs1*, and *hadh*, all of which were metabolism-related.

Table 6 provides a partial list of the DEGs in the liver tissue of larvae fed Art or MD. DEGs were divided into 4 categories: lipid metabolism, protein metabolism, mitochondria synthesis, and ribosome synthesis. Gene name, description, read count, and Log_2_-fold change, as well as *P*-adj values, are presented. All DEGs are shown in Table S4.

### 3.6. Multidimensional Correlation Analysis

**Figure 4:**
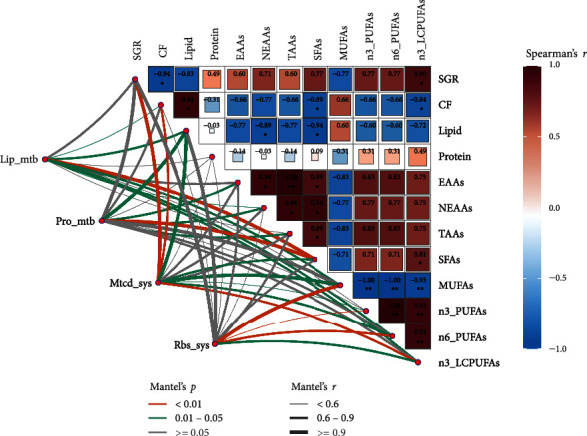
Multidimensional correlation analysis between gene expression and larval nutrition parameters. Nutritional parameters: specific growth rate (SGR); larval body lipid, protein, and essential amino acid (EAA) contents; nonessential amino acid (NEAA) content; total amino acid (TAA) content; saturated fatty acid (SFA) proportion monounsaturated fatty acid (MUFA) proportion; n-3 polyunsaturated fatty acid (n3_PUFA) proportion; and n-6 polyunsaturated fatty acid (n6_PUFA). Gene expression: liver tissue protein metabolism-related genes (Pro_mtb), lipid metabolism-related genes (Lip_mtb), mitochondria synthesis-related genes (Mtcd_sys), and ribosome synthesis-related genes (Rbs_sys) are listed in Table 6. Nutritional parameter comparisons in pairs are shown, with Pearson's correlation coefficients indicated by a color gradient and color block size. Mantel's tests show a correlation between related gene expression levels and defined factors. Mantel's *r* statistic and Mantel's *p* statistic for the relevant distance correlations, respectively, are represented by edge width and color.

As seen in Figure 4, a correlation study between nutritional factors that affect growth revealed a strong positive link between the SGR and the body's n-3 LC-PUFA proportion (*P* < 0.05). The body NEAA, EAA, and TAA contents were generally coordinated with the body LC-PUFA proportion. The body MUFA proportion was significantly negatively correlated with PUFAs (*P* < 0.01). Lipid and protein metabolism were found to be considerably closely associated to the total body SFA fraction by correlation analysis between liver tissue gene expression and body parameters. (Mantel's *r* ≥ 0.9, *P* < 0.01). Gene expression associated with mitochondrial biosynthesis was significantly correlated with SGR (Mantel's *r* ≥ 0.9, *P* < 0.01). In addition, mitochondrial biosynthesis- (Mantel's *r* ≥ 0.9, *P* < 0.05) and ribosome biosynthesis- (Mantel's *r* ≥ 0.9, *P* < 0.01) related gene expression was significantly strongly correlated with the larval body MUFA proportion.

## 4. Discussion

Our previous study revealed the indispensable role of PLs in promoting growth performance and decreasing mortality in the larval stage of this species, principally by improving intestinal morphology and PL catabolism, which increases food absorption while saving amino acids for protein synthesis [20]. Balanced nutrition should be designed to ensure adequate PLs for fish early life stages. We estimated the optimal larval SL requirement in MD form; however, SL enrichment protocols have not been studied.

### 4.1. Appropriate SL Enrichment Protocol in *Artemia* Nauplii

*Artemia* nauplii intake the enrichment product by filtration; therefore, slight changes in the enrichment parameters may affect the naupliar filtering capacity, ultimately affecting the composition of the enriched *Artemi*a nauplii. Previous studies have reported that it is feasible to enrich PLs using SL at a dose of 0.6 g/L for 24 h [28]. In line with this, *Artemia* nauplii PL content increased with increased SL dosage and enrichment time in an interactive mode. The PL content of live prey appeared to be unaffected by an SL dosage of 5 g/m^3^, whereas 10 g and 20 g/m^3^ SL resulted in similar PL contents. In the time dimension, the SL content was significantly increased from 6 h to 12 h and 12 h to 24 h. SL enrichment for 24 h resulted in the highest PL content in live food, while the value was comparable to that at 12 h. The appropriate SL strategy should balance other nutrients, such as protein, lipids, fatty acids, and amino acids, as their indispensable roles as food. Analyses indicated that *Artemia* enrichment time significantly decreased live prey protein content, n-3 LC-PUFA proportion, and TAA content, indicating that excessive enrichment time could decrease live food nutritional value. Moreover, we determined the body length of *Artemia* nauplii and found a significantly larger size after 24 h of enrichment (0 h: 579.99 ± 6.27 versus 24 h: 812.65 ± 15.22 *μ*m, *n* = 10, *P* < 0.05). By balancing PL enrichment and nutrient catabolism, as well as live food particle size, it is proposed that 12 h enrichment with 10 g/m^3^ SL could be considered an optimal enrichment protocol. Previous studies have shown that it is possible to increase the levels of PLs and n-3 LC-PUFAs within the PL fraction of *Artemia* nauplii using marine lecithin [17, 28, 29]. Nevertheless, SL contains good PLs (mainly PC) irrespective of n-3 LC-PUFAs and is 10 to 30 times cheaper than marine lecithin, which may still be of benefit to hatcheries.

### 4.2. Feeding with MD Promotes Larval Growth Performance at the Late Larval Stage

Studies have been carried out to investigate weaning strategies by adjusting the live food/MD ratio, feeding frequency, and onset weaning time [7]. Survival rate results showed sudden weaning onto the MD showed no significant effect on the mortality of larvae (40 dph), which indicated that this species can easily acclimatize to accept MD at an early stage [30]. The investigation of larval growth performance could approximately evaluate the appropriate feeding regimes. The simple rationale behind this overwhelming choice is that more appropriate formulations or rearing strategies will support optimal growth rates and, conversely, deficient ones will be less successful in supporting normal growth [31]. In the present study, larvae fed live prey showed significantly higher growth performance than larvae fed MD at end stage 1 (larval 25–40 dph). The growth performance promotion effects could generally be attributed to the immature digestive system for MD and nutritional factors (e.g., free amino acids and phospholipids) in *Artemia* nauplii, which could stimulate digestive enzyme secretions or enhance ingestion by synergistic visual or chemical stimulations of the larvae [32–34]. Another interesting finding is that larvae fed the MD showed better growth performance than larvae fed the live prey at stage 2 (41–55 dph). At the end of this stage, MD completely reversed the retarded growth performance, reaching comparable values with the live prey group. The abovementioned success of feeding with the MD was probably due to the high-density nutrient provision by the MD and well-developed digestive system for MD digestion, as well as better ingestion by well-developed swimming ability at this stage. These findings suggest that feeding with MD promotes larval growth performance at the late larval stage. It is foreseeable that better growth performance can be achieved by cofeeding the larvae with live prey and MD at stage 1 and then progressively replacing live prey with MD at stage 2.

### 4.3. Liver Tissue Metabolism and Immunity Diversity in Larvae Fed Live Prey or MD

To elucidate the possible mechanisms underlying the growth promotion effect of feeding with MD at stage 2, biochemical analysis and RNA sequencing were carried out. The biomass increase was primarily attributed to lipid and protein anabolism, and the growth promotion effect could be attributed to the diversity of protein and lipid catabolism at the enzyme and mRNA expression levels. In this section, lipid and protein metabolism are discussed.

#### 4.3.1. Feeding with MD on Larval Liver Tissue Lipid Metabolism

Among the 10 hub genes predicted by String, 8 were directly related to lipid metabolism. RNA sequencing results indicated that MD primarily increased the expression of lipogenesis-related genes, such as *acaca*, *acsbg2*, *elovl5*, *fads2*, *hmgcr*, and *srebp1*. Genes *acaca* and *hmgcr* encoding proteins are associated with lipogenesis and cholesterol synthesis, respectively. ACACA is the rate-limiting enzyme of the first step in de novo lipid synthesis and catalyzes the irreversible carboxylation of acetyl-CoA to produce malonyl-CoA. Malonyl-CoA can either be incorporated into fatty acid synthesis or act allosterically to inhibit activated fatty acid oxidation in the mitochondria [35, 36]. The *hmgcs1* gene encodes a protein that catalyzes the condensation of acetyl-CoA with acetoacetyl-CoA to form HMG-CoA, which is converted by HMGCR into mevalonate, a precursor for cholesterol synthesis [37]. Upregulation of these genes could be regulated by *srebp1*, which encodes a key transcription factor that regulates the expression of genes involved in cholesterol biosynthesis and lipid homeostasis [38]. Notably, the MD significantly increased PL key catabolism-related *pla2g3* [39], *lpin1* [40], and *plpp1* [41] gene expression but conversely decreased PL anabolism-related *pemt* [42] gene expression. A crowding-out effect caused by high dietary SL inclusion is characterized by increased PL degradation and increased lipid biosynthesis, as observed in our previous study [20]. According to these data, we can infer that a higher phospholipid transfer effect can be obtained by feeding with SL-enriched MD rather than SL-enriched *Artemia* nauplii.

There were somewhat surprising findings in which the expression levels of genes are associated with mitochondrial biogenesis (*mrpl37*, *mrps30*, and *mrpl9*). Additionally, genes associated to mitochondrial intermembrane chaperones (*tim8a*, *tim10*, and *tim17a*) were markedly elevated. Due to the preference for PLs as an energy source throughout the larval stage, mitochondrial biogenesis adjusts to PL catabolism [43]. The priority of PL catabolism could lead to decreased whole-body lipid content, as the fat-reducing effect was widely observed in a PL-rich diet [44]. Analysis of the multidimensional association between gene expression and nutritional factors for larvae indicated the whole-body lipid content, which could provide additional proof for this speculation.

#### 4.3.2. Feeding with MD on Larval Liver Tissue Protein Metabolism

Protein metabolism is intimately tied to individual growth, or more specifically, protein retention, and is predominantly carried out in the ribosome. Aspartate aminotransferase (AST) and alanine aminotransferase (ALT) are the key enzymes mediating AA catabolism through the irreversible transamination of AA into TCA cycle precursors [45, 46]. According to a prior study, protein retention was adversely linked with ALT and AST activity [47]. Additionally, largemouth bass (*Micropterus salmoides*) larvae's AST and ALT activity was dramatically lowered by the marine protein hydrolysate's inclusion in the meal, which also demonstrated a growth-promoting effect [48]. The activity of the enzymes involved in amino acid catabolism, ALT and AST, in liver tissue was drastically lowered in larvae fed the MD diet, demonstrating that the larvae fed the MD diet had a lower flow of amino acids into the TCA cycle for the generation of ATP. Significantly higher NEAA, EAA, and TAA contents were observed in live prey than those in the MD, while significantly higher parameters were observed in larvae fed with the MD. The discrepancy in results could be partly attributed to the ALT and AST enzyme activity diversity in liver tissue.

Protein turnover is an ATP-cost bioprocess, and enhanced protein synthesis is usually accompanied by decreased amino catabolism. Feeding with the MD significantly increased the expression of genes encoding protein translation initiation factors, such as *eif4e* and *eif4g1*. The translation factor eIF4E, which is central to protein synthesis in general, recognizes and binds mRNA caps during an early step in the initiation of protein synthesis [49]. The initiation factor eIF4G plays a central role in translation initiation through its interactions with the cap-binding protein eIF4E [50]. The increased expression of *eif4e* and *eif4g1* combined with decreased expression of *nelfa*, a complex that negatively regulates the elongation of transcription [51], could indicate increased protein synthesis. The biogenesis of ribosomes is an ATP-costly process; thus, cells tightly control RP expression [52]. In this study, feeding with MD significantly enhanced expression of the ribosome biogenesis-related genes *gfm1*, *rrbp1*, *bop1*, *brix1*, *bms1*, and *rrs1* in the liver tissue. These protein-coding genes are crucial for the processes of ribosome biosynthesis, maturation, and assembly [53–56]. Taken together, at feeding stage 2, MD decreased larval amino acid catabolism to TCA but increased protein translation and ribosome biosynthesis compared with those of larvae fed with live prey. Therefore, in the MD group, the elevated biomass could be the cause of the elevated amino acid synthesis and lower amino acid catabolism. According to a multidimensional correlation analysis between gene expression and larval feeding parameters, growth performance was substantially associated with fatty acid composition, especially the n-3 LC-PUFA proportion and mitochondrial biosynthesis-related gene expression, rather than protein metabolism. Nonetheless, protein metabolism was closely correlated with larval fatty acid profiles, indicating the close relationship between protein and lipid metabolism.

#### 4.3.3. Feeding with MD on Larval Liver Tissue Immunity

Surprisingly, results showed feeding with MD significantly decreased proinflammatory genes expression, such as *cd68*, *tlr5*, *il4*, *ccl25*, *tnfsf10*, *il6st*, and *irf6*. These results supported the idea that n-3 LC-PUFAs exerted anti-inflammatory effects in fish species [23, 25]. In line with that significantly higher n-3 LC-PUFA proportion was found in larval body (Table 5). Another possible explanation for this might be that live prey can carry a high load of bacteria, including opportunistic bacteria namely *Vibrio* spp. that may cause larval inflammatory response, diseases, and mass mortality episodes [57]. Thus, the substitution of live prey by MD at optimal time point can beneficial to larval health.

## 5. Conclusions

To establish an appropriate rearing strategy for rock bream, the present study utilized an enrichment protocol regarding SL dosage and time and compared the growth performance and liver tissue metabolism in larvae reared on soybean lecithin-enriched *Artemia* nauplii or MD.

1. SL can be successfully enriched in *Artemia* nauplii. Seawater (10 g SL/m^3^) and 12 h can be regarded as the appropriate enrichment protocol
2. Retard weaning onto MD led to lower growth performance at the late larval stage (41–55 dph)
3. Larval growth promotion by feeding with MD was probably attributed to increased lipogenesis and protein synthesis

## Figures and Tables

**Table 1:** Proximate nutrient composition analysis of *Artemia* nauplii enriched with soybean lecithin for different durations and different dosages.

Dosage (g/m^3^)	Time (h)	PLs content (%, DW)	Protein (%, DW)	Lipid (%, DW)	Moisture (%, TW)	Ash (%, DW)
0	0	13.35 ± 1.45^ABC^	58.09 ± 0.32^z^	21.16 ± 0.28^abcd^	80.40 ± 0.23^v^	6.83 ± 0.12^A^
0	6	11.94 ± 3.26^AB^	55.56 ± 1.13^y^	18.46 ± 4.26^abcd^	83.26 ± 0.61^wxy^	8.65 ± 0.74^AB^
0	12	10.73 ± 2.64^AB^	55.54 ± 0.31^y^	18.08 ± 5.20^abc^	83.29 ± 0.84^wxy^	10.57 ± 0.18^BC^
0	24	9.39 ± 2.54^A^	54.22 ± 1.33^y^	14.43 ± 3.95^a^	85.79 ± 2.97^yz^	11.26 ± 2.48^BC^
5	6	12.08 ± 2.60^AB^	55.25 ± 1.32^y^	22.01 ± 0.45^bcd^	82.63 ± 0.93^w^	8.65 ± 0.43^AB^
5	12	11.72 ± 4.78^AB^	54.93 ± 0.53^y^	18.94 ± 6.34^abcd^	83.72 ± 0.49^wxy^	9.86 ± 0.75^BC^
5	24	15.46 ± 2.32^ABC^	51.55 ± 1.90^x^	16.07 ± 5.88^ab^	85.51 ± 1.93^xyz^	14.08 ± 1.11^D^
10	6	16.22 ± 2.47^BC^	54.45 ± 1.30^y^	23.38 ± 1.82^cd^	83.02 ± 1.20^wx^	8.57 ± 0.42^AB^
10	12	22.91 ± 4.72^DE^	54.59 ± 1.37^y^	23.74 ± 3.01^cd^	83.70 ± 0.59^wxy^	10.23 ± 0.91^BC^
10	24	27.22 ± 3.84^E^	51.31 ± 2.37^x^	24.96 ± 4.41^d^	86.37 ± 0.86^z^	16.23 ± 2.29^D^
20	6	18.35 ± 0.46^CD^	54.84 ± 1.76^y^	22.71 ± 1.84^bcd^	83.19 ± 1.56^wxy^	8.76 ± 0.33^AB^
20	12	22.82 ± 1.93^DE^	55.26 ± 0.59^y^	24.18 ± 3.62^cd^	84.29 ± 1.63^wxyz^	10.02 ± 1.05^BC^
20	24	28.54 ± 4.80^E^	51.51 ± 2.27^x^	24.13 ± 3.33^cd^	86.44 ± 1.24^z^	15.82 ± 2.43^D^

Two-way	Dosage	<0.001	0.123	<0.001	0.749	0.090
ANOVA	Time	<0.001	<0.001	0.648	<0.001	<0.001
*P*	*D* × *T*	<0.001	0.693	0.233	0.996	0.011

Superscript letters a, b, and c indicate the SL dosage effect. Superscript letters x, y, and z represent the SL time effect. Superscript letters A, B, and C represent the SL dosage and time interaction effect. Values (mean ± S.E.M., *n* = 3) without superscript letters or sharing the same superscript within a column indicate no significant difference (*P* > 0.05).

**Table 2:** Fatty acid composition analysis of *Artemia* nauplii enrichment.

Dosage (g/m^3^)	Time (h)	SFAs^1^ (%)	MUFAs^2^ (%)	n-3 PUFAs^3^ (%)	n-6 PUFAs^4^ (%)	n-3 LC-PUFAs^5^ (%)
0	0	15.35 ± 0.21^x^	42.71 ± 0.15^x^	29.69 ± 0.68	11.45 ± 0.29	16.86 ± 0.47^z^
0	6	16.41 ± 0.43^y^	43.83 ± 0.93^x^	27.88 ± 0.62	11.12 ± 0.15	16.12 ± 1.07^yz^
0	12	15.39 ± 0.08^x^	43.42 ± 0.21^x^	29.21 ± 0.40	11.19 ± 0.09	16.34 ± 0.00^yz^
0	24	16.45 ± 0.54^y^	46.10 ± 0.28^y^	27.07 ± 1.54	10.01 ± 0.35	13.22 ± 0.19^w^
5	6	15.86 ± 0.90^xy^	43.40 ± 1.97^x^	28.64 ± 2.30	11.34 ± 0.51	16.19 ± 1.77^yz^
5	12	15.59 ± 0.28^xy^	43.62 ± 0.40^x^	28.69 ± 0.87	11.33 ± 0.18	16.03 ± 0.31^yz^
5	24	15.98 ± 0.12^xy^	44.19 ± 0.63^x^	27.81 ± 0.63	11.28 ± 0.07	15.69 ± 0.97^xyz^
10	6	15.56 ± 0.07^xy^	42.68 ± 0.15^x^	29.42 ± 0.18	11.6 ± 0.12	16.78 ± 0.15^z^
10	12	16.00 ± 0.33^xy^	44.09 ± 0.63^x^	27.77 ± 0.74	11.40 ± 0.27	15.44 ± 0.77^xyz^
10	24	16.05 ± 0.56^xy^	43.96 ± 0.96^x^	28.25 ± 2.28	11.02 ± 0.82	14.59 ± 0.47^wxy^
20	6	15.72 ± 0.39^xy^	42.66 ± 1.33^x^	29.26 ± 1.33	11.61 ± 0.34	16.40 ± 1.14^z^
20	12	15.77 ± 0.33^xy^	43.23 ± 0.45^x^	28.65 ± 0.47	11.83 ± 0.43	15.88 ± 0.30^yz^
20	24	16.48 ± 0.52^y^	44.01 ± 0.18^x^	27.47 ± 1.71	11.54 ± 1.35	14.12 ± 0.96^wx^

Two-way	Dosage	0.836	0.349	0.969	0.212	0.678
ANOVA	Time	0.015	0.005	0.091	0.229	<0.001
*P*	*D* × *T*	0.543	0.578	0.891	0.844	0.444

Superscript letters a, b, and c indicate the SL dosage effect. Superscript letters x, y, and z represent the SL time effect. Superscript letters A, B, and C represent dosage and time interaction effects. Values (mean ± S.E.M., *n* = 3) without superscript letters or sharing the same superscript within a column indicate no significant difference (*P* > 0.05). ^1^SFAs: saturated fatty acids. ^2^MUFAs: monounsaturated fatty acids. ^3^n-3 PUFAs: n-3 polyunsaturated fatty acids. ^4^n-6 PUFAs: n-6 polyunsaturated fatty acids. ^5^n-3 LC-PUFAs: n-3 long-chain polyunsaturated fatty acids.

**Table 3:** Amino acid composition analysis of live prey enrichment.

Dosage (g/m^3^)	Time (h)	TAAs^1^	EAAs^2^	NEAAs^3^
0	0	43.60 ± 0.24^d,z^	21.34 ± 0.31^z^	22.26 ± 0.38^F^
0	6	40.39 ± 0.46^bc,xy^	19.86 ± 0.26^y^	20.53 ± 0.72^B^
0	12	42.09 ± 1.14^cd,yz^	20.24 ± 0.59^y^	21.86 ± 0.55^EF^
0	24	42.05 ± 1.70^cd,yz^	19.91 ± 1.07^y^	22.14 ± 0.63^F^
5	6	41.64 ± 1.15^bc,xy^	20.20 ± 0.64^y^	21.44 ± 0.57^BCDEF^
5	12	41.01 ± 0.94^bc,xy^	19.90 ± 0.55^y^	21.11 ± 0.40^BCDE^
5	24	40.67 ± 0.48^bc,xy^	19.56 ± 0.52^y^	21.11 ± 0.05^BCDE^
10	6	40.85 ± 1.25^bc,xy^	19.79 ± 0.78^y^	21.06 ± 0.48^BCDE^
10	12	40.04 ± 0.45^b,x^	19.29 ± 0.53^xy^	20.75 ± 0.25^BCD^
10	24	37.80 ± 0.55^a,w^	18.23 ± 0.13^x^	19.57 ± 0.44^A^
20	6	42.02 ± 0.62^cd,yz^	20.29 ± 0.55^y^	21.73 ± 0.35^DEF^
20	12	41.51 ± 0.85^bc,xy^	19.95 ± 0.52^y^	21.56 ± 0.33^CDEF^
20	24	39.86 ± 0.90^b,x^	19.16 ± 0.32^xy^	20.70 ± 0.58^BC^

Two-way	Dosage	0.027	0.123	0.017
ANOVA	Time	<0.001	<0.001	<0.001
*P*	*D* × *T*	0.060	0.648	0.006

Superscript letters a, b, and c indicate the SL dosage effect. Superscript letters x, y, and z represent the SL time effect. Superscript letters A, B, and C represent dosage and time interaction effects. Values (mean ± S.E.M., *n* = 3) without superscript letters or sharing the same superscript within a column indicate no significant difference (*P* > 0.05). ^1^TAAs: total amino acids. ^2^EAAs: essential amino acids. ^3^NEAAs: nonessential amino acids.

**Table 4:** Growth performance and body indices of 40 dph and 55 dph rock bream larvae.

Stages	Parameters	Larvae fed on different food sources	
		Art	MD
Stage 1	Standard length (cm)	1.76 ± 0.04	1.60 ± 0.05
	BW (g)	0.20 ± 0.03^∗^	0.13 ± 0.02
	WGR (%)	1145.3 ± 175.2^∗^	736.3 ± 79.7
	SGR (%/d)	15.59 ± 0.74	15.10 ± 0.72

Stage 2	Standard length (cm)	3.48 ± 0.05	3.73 ± 0.06^∗∗^
	BW (g)	1.46 ± 0.04	1.50 ± 0.05
	WGR (%)	653.0 ± 24.16	1087.9 ± 42.33^∗∗∗^
	SGR (%/d)	12.50 ± 0.24	15.46 ± 0.23^∗∗∗^

BW: body weight; WGR: weight gain rate; SGR: specific growth rate. Values (mean ± S.E.M., *n* = 3) within a row with ^∗^*P* < 0.05, ^∗∗^*P* < 0.01, and ^∗∗∗^*P* < 0.001; without ^∗^ denotes no significant difference (*P* > 0.05).

**Table 5:** Proximate nutrient composition, fatty acid composition, and amino acid content analysis of the larval body and their correlation analysis with food sources.

Nutrients	Food sources		Larval body	
	Live prey	Diet	Art	MD
Crude protein (%)	54.59	58.95	15.25 ± 0.02	15.26 ± 0.07
Crude lipid (%)	23.74	18.58	5.77 ± 0.01^∗∗∗^	3.99 ± 0.02
Ash (%)	10.23	10.16	3.57 ± 0.02	3.72 ± 0.04^∗^
Moisture (%)	83.71	9.55	74.76 ± 0.19	75.94 ± 0.29^∗^
SFAs (%)	16.00	27.44	28.21 ± 0.06	50.73 ± 2.36^∗∗∗^
MUFAs (%)	44.09	18.18	68.67 ± 1.08^∗∗∗^	31.72 ± 1.61
n-3 PUFAs (%)	27.77	12.01	0.31 ± 0.01	2.80 ± 0.77^∗^
n-6 PUFAs (%)	11.40	41.84	2.40 ± 0.93	14.31 ± 2.89^∗^
n-3 LC-PUFAs (%)	15.44	7.79	0.14 ± 0.01	2.17 ± 0.45^∗∗^
EAAs (mg/g)	19.29	18.95	23.39 ± 0.34	25.31 ± 0.18^∗∗^
NEAAs (mg/g)	20.75	18.53	27.06 ± 0.41	29.55 ± 0.52^∗^
TAAs (mg/g)	40.04	38.04	50.45 ± 0.75	54.86 ± 0.70^∗^

Correlation (*R*^2^, *P*)	Live prey × art		Diet × MD	
Fatty acids	0.61, <0.001		0.62, <0.001	
Amino acids	1.00, <0.001		1.00, <0.001	
Proximate nutrients	0.81, <0.001		0.07, ns	

Values (mean ± S.E.M., *n* = 3) within a row with ^∗^*P* < 0.05, ^∗∗^*P* < 0.01, and ^∗∗∗^*P* < 0.001; without ^∗^ denotes no significant difference (*P* > 0.05).

**Table 6:** A partial list of the differentially expressed genes (DEGs) in liver tissue of larvae fed art or MD.

Gene name	Description	Art read count	MD read count	Log_2_-fold change	*P*-adj
Lipid metabolism					
*hmgcr*	3-Hydroxy-3-methylglutaryl-coenzyme A reductase	223.31	551.14	-1.30	1.54*E* − 04
*acaca*	Acetyl-CoA carboxylase	116.89	2424.06	-4.37	6.00*E* − 03
*elovl5*	Elongation of very long-chain fatty acid protein 5	111.17	526.96	-2.24	6.39*E* − 12
*fads2*	Fatty acid desaturase 2	6.11	935.96	-7.26	4.45*E* − 04
*hmgcs1*	Hydroxymethylglutaryl-CoA synthase	870.00	2022.97	-1.22	5.38*E* − 03
*lpin1*	Phosphatidate phosphatase LPIN1	157.95	2725.27	-4.11	6.61*E* − 03
*plpp1*	Phospholipid phosphatase 1	29.80	89.93	-1.59	9.81*E* − 04
*srebp1*	Sterol regulatory element-binding protein 1	151.61	648.52	-2.10	3.20*E* − 04
*acsbg2*	Long-chain-fatty-acid-CoA ligase ACSBG2	217.96	894.54	-2.04	1.54*E* − 04
*pla2g3*	Group 3 secretory phospholipase A2	220.31	856.43	-1.96	2.15*E* − 02
*pemt*	Phosphatidylethanolamine N-methyltransferase	596.47	167.95	1.83	4.53*E* − 11

Protein metabolism					
*eif4e*	Eukaryotic translation initiation factor 4E	349.79	742.94	-1.09	2.82*E* − 03
*eif4g1*	Eukaryotic translation initiation factor 4 gamma 1	432.35	1209.11	-1.48	3.12*E* − 06
*eif4e2*	Eukaryotic translation initiation factor 4E type 2	136.29	64.91	1.07	2.19*E* − 02
*eif6*	Eukaryotic translation initiation factor 6	295.68	572.38	-0.95	1.52*E* − 02
*nelfa*	Negative elongation factor A	886.50	505.01	0.81	4.35*E* − 02

Mitochondria synthesis					
*timm10*	Translocase subunit Tim 10	68.27	169.86	-1.32	1.92*E* − 03
*timm17a*	Translocase subunit Tim 17A	202.44	368.43	-0.86	3.82*E* − 02
*timm8a*	Translocase subunit Tim 8A	87.11	173.60	-0.99	3.76*E* − 02
*mrpl37*	39S ribosomal protein L2	93.25	186.86	-1.00	3.09*E* − 02

Ribosome synthesis					
*gfm1*	Elongation factor G	22.76	129.09	-2.50	2.02*E* − 08
*rrbp1*	Ribosome-binding protein 1	75.70	202.33	-1.42	2.30*E* − 03
*bop1*	Ribosome biogenesis protein bop1	115.42	269.69	-1.22	1.94*E* − 03
*brix1*	Ribosome biogenesis protein BRX1 homolog	36.09	95.48	-1.40	4.32*E* − 03
*bms1*	Ribosome biogenesis protein BMS1 homolog	44.64	117.61	-1.40	2.56*E* − 03
*rrs1*	Ribosome biogenesis regulatory protein homolog	74.88	167.94	-1.17	7.16*E* − 03

Immunity					
*cd81*	CD81 antigen	265.79	144.79	0.88	4.01*E* − 02
*ccl25*	C-C motif chemokine 25	191.25	77.83	1.30	2.21*E* − 02
*igg1*	Immunoglobulin gamma-1 heavy chain	271.13	113.01	1.26	1.27*E* − 03
*irf6*	Interferon regulatory factor 6	547.71	248.28	1.14	5.43*E* − 03
*il6st*	Interleukin-6 receptor subunit beta	274.78	29.47	3.22	9.87*E* − 04
*cd68*	Macrosialin	2077.35	942.52	1.14	7.14*E* − 04
*nlrc3*	NLR family CARD domain-containing protein 3	219.01	111.89	0.97	3.10*E* − 02
*nfil3*	Nuclear factor interleukin-3-regulated protein	75.80	15.58	2.28	5.86*E* − 06
*tlr5*	Toll-like receptor 5	280.09	138.44	1.02	1.01*E* − 02
*tnfsf10*	Tumor necrosis factor ligand superfamily member 10	430.13	205.05	1.07	8.03*E* − 03

## Data Availability

The diet formulation, primer sequences, RNA-seq basic information, and DEGs generated by RNA sequencing used to support the findings of this study are included within the supplementary information files.

## Abbreviations

*acaca*: Acetyl-CoA carboxylase
*acadl*: Very long-chain specific acyl-CoA dehydrogenase
*acadm*: Medium-chain specific acyl-CoA dehydrogenase
*acly*: ATP-citrate synthase
*acsbg2*: Long-chain-fatty-acid-CoA ligase ACSBG2
*bms1*: Ribosome biogenesis protein BMS1 homolog
*bop1*: Ribosome biogenesis protein bop1
*brix1*: Ribosome biogenesis protein BRX1 homolog
*ccl25*: C-C motif chemokine 25
*cd68*: CD68 antigen
*cd81*: CD81 antigen
*cox5b*: Cytochrome c oxidase subunit 5B
dph: Days posthatching
*eif4e*: Eukaryotic translation initiation factor 4E
*eif4e2*: Eukaryotic translation initiation factor 4E type 2
*eif4g1*: Eukaryotic translation initiation factor 4 gamma 1
*eif6*: Eukaryotic translation initiation factor 6
*elovl5*: Elongation of very long-chain fatty acid protein 5
*fads2*: Fatty acid desaturase 2
*gfm1*: Elongation factor G
*glud1*: Glutamate dehydrogenase 1
*glul*: Glutamate-ammonia ligase
*got*: Glutamate oxaloacetate transaminase
*hadh*: Hydroxyacyl-coenzyme A dehydrogenase
*hmgcr*: 3-Hydroxy-3-methylglutaryl-coenzyme A reductase
*hmgcs1*: Hydroxymethylglutaryl-CoA synthase
*igg1*: Immunoglobulin gamma-1 heavy chain
*il4*: Interleukin 4
*il6st*: Interleukin-6 receptor subunit beta
*irf6*: Interferon regulatory factor 6
*lpin1*: Phosphatidate phosphatase LPIN1
*mrpl37*: 39S ribosomal protein L2
*mrpl9*: 9S ribosomal protein L9
*mrps30*: 28S ribosomal protein S30
*nelfa*: Negative elongation factor A
*nfil3*: Nuclear factor interleukin-3-regulated protein
*nlrc3*: NLR family CARD domain-containing protein 3
*pemt*: Phosphatidylethanolamine N-methyltransferase
*pla2g3*: Group 3 secretory phospholipase A2
*plpp1*: Phospholipid phosphatase 1
*rrbp1*: Ribosome-binding protein 1
*rrs1*: Ribosome biogenesis regulatory protein homolog
*srebp1*: Sterol regulatory element-binding protein 1
*srebp2*: Sterol regulatory element-binding protein 2
*timm7*: Translocase subunit Tim 7
*timm10*: Translocase subunit Tim 10
*timm23*: Translocase subunit Tim 23
*timm8a*: Translocase subunit Tim 8A
*timm17a*: Translocase subunit Tim 17A
*tlr5*: Toll-like receptor 5
*tnfsf10*: Tumor necrosis factor ligand superfamily member 10
ALT: Alanine aminotransferase
AST: Aspartate aminotransferase
BW: Body weight
DEGs: Differential expression genes
DW: Dry weight
EAAs: Essential amino acids
FAMEs: Fatty acid methyl esters
FBW: Final body weight
GO: Gene Ontology
IBW: Initial body weight
KEGG: Kyoto Encyclopedia of Genes and Genomes
LC-PUFAs: Long-chain polyunsaturated fatty acids
LP: Lipase
MD: Microdiet
MUFAs: Monounsaturated fatty acids
NEAAs: Nonessential amino acids
PBS: Phosphate-buffered saline
PL: Phospholipids
PUFAs: Polyunsaturated fatty acids
SFAs: Saturated fatty acids
SGR: Specific growth rate
SL: Soybean lecithin
SR: Survival rate
TAAs: Total amino acids
TW: Total weight
UMIs: Unique molecular identifiers
WGR: Weight gain rate.

## Supplementary Materials

Supplementary Materials Table S1: formulation and nutrient analysis of microdiets (MD). Table S2: primers used for qRT–PCR validation. Table S3: basic information for the RNA sequencing data and mapping. Table S4: All DEG list.

## References

[1] Cahu C., Infante J. Z. (2001). Substitution of live food by formulated diets in marine fish larvae. *Aquaculure*.

[2] Cahu C. L., Zambonino Infante J. L., Barbosa V. (2003). Effect of dietary phospholipid level and phospholipid: neutral lipid value on the development of sea bass (*Dicentrarchus labrax*) larvae fed a compound diet. *British Journal of Nutrition*.

[3] Engrola S., Figueira L., Conceição L. E. C., Gavaia P. J., Ribeiro L., Dinis M. T. (2009). Co-feeding in senegalese sole larvae with inert diet from mouth opening promotes growth at weaning. *Aquaculture*.

[4] Faulk C. K., Holt G. J. (2009). Early weaning of southern flounder, *Paralichthys lethostigma*, larvae and ontogeny of selected digestive enzymes. *Aquaculture*.

[5] Pradhan P. K., Jena J., Mitra G., Sood N., Gisbert E. (2014). Effects of different weaning strategies on survival, growth and digestive system development in butter catfish *Ompok bimaculatus* (Bloch) larvae. *Aquaculture*.

[6] Rosenlund G., Stoss J., Talbot C. (1997). Co-feeding marine fish larvae with inert and live diets. *Aquaculture*.

[7] Hamre K., Yúfera M., Rønnestad I., Boglione C., Conceição L. E. C., Izquierdo M. (2013). Fish larval nutrition and feed formulation: knowledge gaps and bottlenecks for advances in larval rearing. *Reviews in Aquaculture*.

[8] Kandathil Radhakrishnan D., AkbarAli I., Schmidt B. V., John E. M., Sivanpillai S., Thazhakot Vasunambesan S. (2020). Improvement of nutritional quality of live feed for aquaculture: an overview. *Aquaculture Research*.

[9] Ramos-Llorens M., Ribes-Navarro A., Navarro J. C., Hontoria F., Kabeya N., Monroig Ó. (2023). Can *Artemia franciscana* produce essential fatty acids? Unveiling the capacity of brine shrimp to biosynthesise long-chain polyunsaturated fatty acids. *Aquaculture*.

[10] Carmona-Antoñanzas G., Taylor J. F., Martinez-Rubio L., Tocher D. R. (2015). Molecular mechanism of dietary phospholipid requirement of Atlantic salmon, *Salmo salar* , fry. *Biochimica et Biophysica Acta (BBA) - Molecular and Cell Biology of Lipids*.

[11] Sorgeloos P., Dhert P., Candreva P. (2001). Use of the brine shrimp, *Artemia* spp., in marine fish larviculture. *Aquaculture*.

[12] Jamali H., Ahmadifard N., Noori F., Agh N., Gisbert E. (2018). Improving co-feeding strategies for Neotropical green terror cichlid (*Aequidens rivulatus*) larvae with lecithin-enriched Artemia franciscana nauplii: effects on survival, growth performance and body composition. *Aquaculture Research*.

[13] Koven W. M., Kolkovski S., Tandler A., Kissil G. W., Sklan D. (1993). The effect of dietary lecithin and lipase, as a function of age, on n-9 fatty acid incorporation in the tissue lipids of *Sparus aurata* larvae. *Fish Physiology and Biochemistry*.

[14] Hadas E., Koven W., Sklan D., Tandler A. (2003). The effect of dietary phosphatidylcholine on the assimilation and distribution of ingested free oleic acid (18:1 *n* −9) in gilthead seabream (*Sparus aurata*) larvae. *Aquaculture*.

[15] Niu J., Liu Y. J., Tian L. X. (2008). Effects of dietary phospholipid level in cobia (*Rachycentron canadum*) larvae: growth, survival, plasma lipids and enzymes of lipid metabolism. *Fish Physiology and Biochemistry*.

[16] Gisbert E., Villeneuve L., Zambonino-Infante J. L., Quazuguel P., Cahu C. L. (2005). Dietary phospholipids are more efficient than neutral lipids for long-chain polyunsaturated fatty acid supply in European sea bass *Dicentrarchus labrax* larval development. *Lipids*.

[17] Guinot D., Monroig Ó., Navarro J. C., Varó I., Amat F., Hontoria F. (2013). Enrichment of Artemia metanauplii in phospholipids and essential fatty acids as a diet for common octopus (*Octopus vulgaris*) paralarvae. *Aquaculture Nutrition*.

[18] Alejo A., Tafalla C. (2011). Chemokines in teleost fish species. *Developmental and Comparative Immunology*.

[19] Tan P., Zhu W. L., Zhang P., Wang L. G., Chen R. Y., Xu D. D. (2022). Dietary soybean lecithin inclusion promotes growth, development, and intestinal morphology of yellow drum (*Nibea albiflora*) larvae. *Aquaculture*.

[20] Tan P., Zhang P., Zhang L. (2022). Effects of soybean lecithin on growth performance, intestine morphology, and liver tissue metabolism in rock bream (*Oplegnathus fasciatus*) larvae. *Frontiers in Marine Science*.

[21] Association of Official Analytical Chemists (2000). Official Methods of Analysis of AOAC International.

[22] Folch J., Lees M., Sloane Stanley G. H. (1957). A simple method for the isolation and purification of total lipides from animal tissues. *Journal of Biological Chemistry*.

[23] Zuo R., Ai Q., Mai K. (2012). Effects of dietary n-3 highly unsaturated fatty acids on growth, nonspecific immunity, expression of some immune related genes and disease resistance of large yellow croaker (*Larmichthys crocea*) following natural infestation of parasites (*Cryptocaryon irritans*). *Fish & Shellfish Immunology*.

[24] Wu X., Wang L., Xie Q., Tan P. (2020). Effects of dietary sodium butyrate on growth, diet conversion, body chemical compositions and distal intestinal health in yellow drum (*Nibea albiflora*, Richardson). *Aquaculture Research*.

[25] Tan P., Wabike E. E., Qin G. (2020). Effects of dietary n-3 long-chain polyunsaturated fatty acids (n-3 LC-PUFAs) on growth performance, body composition and subcutaneous adipose tissue transcriptome analysis of juvenile yellow drum (*Nibea albiflora*). *Aquaculture Nutrition*.

[26] Livak K. J., Schmittgen T. D. (2001). Analysis of relative gene expression data using real-time quantitative PCR and the 2^−*ΔΔ*C_^_T_ method. *Methods*.

[27] Shannon P., Markiel A., Ozier O. (2003). Cytoscape: a software environment for integrated models of biomolecular interaction networks. *Genome Research*.

[28] Cavrois Rogacki T., Davie A., King E., Esnault S., Migaud H., Monroig O. (2019). Short-term lecithin enrichments can enhance the phospholipid and DHA contents of the polar lipid fraction of *Artemia* nauplii. *Aquaculture*.

[29] Guinot D., Monroig Ó., Hontoria F., Amat F., Varó I., Navarro J. C. (2013). Enriched on-grown *Artemia* metanauplii actively metabolise highly unsaturated fatty acid-rich phospholipids. *Aquaculture*.

[30] Mozanzadeh M. T., Bahabadi M. N., Morshedi V., Azodi M., Agh N., Gisbert E. (2021). Weaning strategies affect larval performance in yellowfin seabream (*Acanthopagrus latus*). *Aquaculture*.

[31] Holt G. J. (2011). Larval fish nutrition.

[32] Kolkovski S. (2001). Digestive enzymes in fish larvae and juveniles--implications and applications to formulated diets. *Aquaculture*.

[33] Kolkovski S., Allan G., Burnell G. (2013). Microdiets as alternatives to live feeds for fish larvae in aquaculture: improving the efficiency of feed particle utilization. *Advances in aquaculture hatchery technology*.

[34] Engrola S., Conceição L. E. C., Dias L., Pereira R., Ribeiro L., Dinis M. T. (2007). Improving weaning strategies for senegalese sole: effects of body weight and digestive capacity. *Aquaculture Research*.

[35] Bianchi A., Evans J. L., Iverson A. J., Nordlund A. C., Watts T. D., Witters L. A. (1990). Identification of an isozymic form of acetyl-CoA carboxylase. *The Journal of Biological Chemistry*.

[36] Munday M. R. (2002). Regulation of mammalian acetyl-CoA carboxylase. *Biochemical Society Transactions*.

[37] Rokosz L. L., Boulton D. A., Butkiewicz E. A. (1994). Human cytoplasmic 3-hydroxy-3-methylglutaryl coenzyme a synthase: expression, purification, and characterization of recombinant wild-type and cys^129^ mutant enzymes. *Archives of Biochemistry and Biophysics*.

[38] Amemiya-Kudo M., Shimano H., Hasty A. H. (2002). Transcriptional activities of nuclear srebp-1a, -1c, and -2 to different target promoters of lipogenic and cholesterogenic genes. *Journal of Lipid Research*.

[39] Murakami M., Masuda S., Shimbara S. (2003). Cellular arachidonate-releasing function of novel classes of secretory phospholipase A_2_s (groups III and XII). *The Journal of Biological Chemistry*.

[40] Han G. S., Carman G. M. (2010). Characterization of the human *LPIN1*-encoded phosphatidate phosphatase isoforms. *The Journal of Biological Chemistry*.

[41] Zhao Y., Kalari S. K., Usatyuk P. V. (2007). Intracellular generation of sphingosine 1-phosphate in human lung endothelial cells: role of lipid phosphate phosphatase-1 and sphingosine kinase 1. *The Journal of Biological Chemistry*.

[42] Shields D. J., Lehner R., Agellon L. B., Vance D. E. (2003). Membrane topography of human phosphatidylethanolamine *N* -methyltransferase. *The Journal of Biological Chemistry*.

[43] Tocher D. R., Bendiksen E. Å., Campbell P. J., Bell J. G. (2008). The role of phospholipids in nutrition and metabolism of teleost fish. *Aquaculture*.

[44] Cai Z., Mai K., Ai Q. (2017). Regulation of hepatic lipid deposition by phospholipid in large yellow croaker. *The British Journal of Nutrition*.

[45] Melo J. F. B., Lundstedt L. M., Metón I., Baanante I. V., Moraes G. (2006). Effects of dietary levels of protein on nitrogenous metabolism of *Rhamdia quelen* (Teleostei: Pimelodidae). *Comparative Biochemistry and Physiology Part A: Molecular & Integrative Physiology*.

[46] Sá R., Pousão-Ferreira P., Oliva-Teles A. (2006). Effect of dietary protein and lipid levels on growth and feed utilization of white sea bream (*Diplodus sargus*) juveniles. *Aquaculture Nutrition*.

[47] He Y., Song H. L., Li G. X. (2010). The relationship of CPS-I, OCT and hepatic encephalopathy. *Zhonghua Gan Zang Bing Za Zhi*.

[48] Dai M., Li S. L., Fu C. F., Qiu H. J., Chen N. S. (2020). The potential role of marine protein hydrolyzates in elevating nutritive values of diets for largemouth bass, *Micropterus salmoides*. *Frontiers in Marine Science*.

[49] Tomoo K., Matsushita Y., Fujisaki H. (2005). Structural basis for mRNA cap-binding regulation of eukaryotic initiation factor 4e by 4e-binding protein, studied by spectroscopic, x-ray crystal structural, and molecular dynamics simulation methods. *Biochimica et Biophysica Acta*.

[50] Nicaise V., Gallois J. L., Chafiai F. (2007). Coordinated and selective recruitment of eif4e and eif4g factors for potyvirus infection in Arabidopsis thaliana. *FEBS Letters*.

[51] Yamaguchi Y., Takagi T., Wada T. (1999). NELF, a multisubunit complex containing RD, cooperates with DSIF to repress RNA polymerase ii elongation. *Cell*.

[52] Piazzi M., Bavelloni A., Gallo A., Faenza I., Blalock W. L. (2019). Signal transduction in ribosome biogenesis: a recipe to avoid disaster. *International Journal of Molecular Sciences*.

[53] Hayano T., Yanagida M., Yamauchi Y., Shinkawa T., Isobe T., Takahashi N. (2003). Proteomic analysis of human Nop56p-associated pre-ribosomal ribonucleoprotein complexes:. *The Journal of Biological Chemistry*.

[54] Sloan K. E., Bohnsack M. T., Watkins N. J. (2013). The 5S RNP couples p53 homeostasis to ribosome biogenesis and nucleolar stress. *Cell Reports*.

[55] Rohrmoser M., Hölzel M., Grimm T. (2007). Interdependence of Pes1, Bop1, and WDR12 controls nucleolar localization and assembly of the PeBoW complex required for maturation of the 60s ribosomal subunit. *Molecular and Cellular Biology*.

[56] Michalec B., Krokowski D., Grela P. (2010). Subcellular localization of ribosomal P0-like protein MRT4 is determined by its n-terminal domain. *The International Journal of Biochemistry & Cell Biology*.

[57] Vadstein O., Bergh Ø., Gatesoupe F.-J. (2013). Microbiology and immunology of fish larvae. *Reviews in Aquaculture*.

