# Supplementary material for: A Comparative Study on Growth Performance, Body Composition, and Liver Tissue Metabolism Rearing on Soybean Lecithin-Enriched Artemia Nauplii and Microdiet in Rock Bream (Oplegnathus fasciatus) Larvae
Source: Aquac Nutr. 2023 Mar 16;2023:5545898. doi: 10.1155/2023/5545898 (PMC10036177; doi:10.1155/2023/5545898)
Supplement: Supplementary Materials — Table S1: formulation and nutrient analysis of microdiets (MD). Table S2: primers used for qRT–PCR validation. Table S3: basic information for the RNA sequencing data and mapping. Table S4: All DEG list. [file 5545898.f1.docx]

**Table S1. Formulation and nutrient analysis of microdiets (MD) (%, dry weight)**

| Ingredients | MD |
| --- | --- |
| Fish meal ^1^ | 21.00 |
| Low-temperature krill meal ^2^ | 14.00 |
| Low-temperature squid meal ^3^ | 12.00 |
| Casein ^4^ | 20.00 |
| Yeast ^5^ | 3.00 |
| Hydrolyzed fish paste ^6^ | 4.00 |
| α-starch ^7^ | 4.00 |
| Sodium alginate | 1.50 |
| Vitamin premix ^8^ | 1.50 |
| Mineral premix ^9^ | 1.50 |
| Ascorbyl polyphosphate | 0.20 |
| Attractant ^10^ | 2.00 |
| Mold inhibitor ^11^ | 0.05 |
| Ethoxyquinine | 0.05 |
| Chloride choline | 0.20 |
| Fish oil | 3.00 |
| SL ^12^ | 12.00 |
| Total | 100.00 |
| *Proximate nutrients* (mean values, % dry weight) | |
| Phospholipids | 12.21 |
| Crude protein | 58.95 |
| Crude lipid | 20.89 |
| Ash | 9.22 |

^1-7^ Fish meal: crude protein, 72.47%, crude lipid, 9.64%; low-temperature krill meal: crude protein, 63.65%, crude lipid, 7.55%; low-temperature squid meal: crude protein, 66.81%, crude lipid, 13.35%; casein: crude protein, 93.62%, crude lipid, 0.76%; yeast: crude protein, 42.6%, crude lipid, 1.00%; hydrolyzed fish paste: 76.92% crude protein, 0.51% crude lipid; α-starch: crude protein, 0.38%, crude lipid, 0.25%.

^8^ Vitamin premix (mg or g kg^-1^ diet): carotene, 0.10 g; vitamin D, 0.05 g; tocopherol, 0.38 g vitamin B_1_, 0.06 g; vitaminB_2_, 0.19 g; vitamin B_6_, 0.05 g; cyanocobalamin, 0.1 mg; biotin, 0.01 g; inositol, 3.85 g; niacin acid, 0.77 g; pantothenic acid, 0.27 g; folic acid, 0.01 g; chloride choline, 7.87 g; and cellulose, 1.92 g.

^9^ Mineral premix (mg or g kg^-1^ diet): NaF, 2 mg; KI, 0.8 mg; CoCl_2_·6H_2_O, 50 mg; CuSO_4_·5H_2_O, 10 mg; FeSO_4_·H_2_O, 80 mg; ZnSO_4_·H_2_O, 50 mg; MnSO_4_·H_2_O, 60 mg; MgSO_4_·7H_2_O, 1200 mg; Ca(H_2_PO_4_)_2_·H_2_O, 3000 mg; NaCl, 100 mg; and zoelite powder, 15.45 g.

^10-12^ Attractant: glycine:betaine = 1:3; mold inhibitor: fumaric acid:calcium propionate = 1:1; SL: Cargill Co., Ltd, Germany, EPIKURON 100G, containing approximately 630 g phospholipids/kg, including 230 g/kg phosphatidylcholine, 190 g/kg phosphatidylethanolamine, 70 g/kg phosphatidylic acid, and 140 g/kg phosphatidylserine.

^13^ Proximate nutrients were presented as the mean to two replicates.

**Table S2. Primers used for qRT‒PCR validation.**

| Primer  names | Primer sequences | TM  (°C) | Product size  (bp) | PCR  Efficiency (%) |
| --- | --- | --- | --- | --- |
| *acaca*-F | GGCTATGTGGGCTCTAGGAG | 60 | 163 | 100 |
| *acaca*-R | GCCAAGCTCATACACGTCAG |  |  |  |
| *hmgcr*-F | GGCTGTCAGTGGGAACTACT | 59 | 160 | 96 |
| *hmgcr*-R | ACTGATGTTCACCTCCACCA |  |  |  |
| *acly* F | CATGAAGAAGCACTCCGACG | 60 | 234 | 102 |
| *acly* R | CCGATCTTAAAACAGCCCGG |  |  |  |
| *eif4e*-F | GAAACCAACCCAAGTCCACC | 58 | 173 | 98 |
| *eif4e*-R | TTGGAGATGAGTCGCAGGTT |  |  |  |
| *acadm*-F | ATCAAGACCCGAGCTGTGAA | 59 | 212 | 97 |
| *acadm*-R | TGGCCCATGTTCATCTCCTT |  |  |  |
| *acadl*-F | AGCAGTTCCTTCGAGAGCTT | 60 | 219 | 97 |
| *acadl*-R | GGTCATGACTGCCAACGATC |  |  |  |
| *nlrc3*-F | TGGATGTGCTGCTGACAAAC | 61 | 239 | 99 |
| *nlrc3*-R | CATGATGTGGAGGCTTCGTG |  |  |  |
| *tlr5*-F | TCTCACATGACAACCTCCCC | 61 | 242 | 98 |
| *tlr5*-R | CAGGAGGTTGGATGACAGGT |  |  |  |
| *ccl25*-F | GCTCCTCACCTGCATGTACT | 57 | 241 | 100 |
| *ccl25*-R | ATTTGCACAGACAGTTCGCT |  |  |  |
| *gas6*-F | TGTTTGCACTCGTTCACCAG | 60 | 152 | 99 |
| *gas6*-R | ACTCTCACCATCACACAGGG |  |  |  |
| *pprc1*-F | CTGCCTCTCCCTCTCCAAAA | 60 | 235 | 98 |
| *pprc1*-R | TAAACATCACTCCACCGCCT |  |  |  |
| *bop1*-F | CAACAGACCGGCAGATAAGC | 59 | 234 | 100 |
| *bop1*-R | TTGTAGGACTCGTGGTGACC |  |  |  |
| *nelfa*-F | TGTATAACGCGAGCAACACG | 60 | 227 | 99 |
| *nelfa*-R | AGCGTACATCTGGTCTCTCG |  |  |  |
| *irf6*-F | ATGAAGAGGTTGGCGAGGAA | 61 | 210 | 101 |
| *irf6*-R | CCACATCCACAGGTTCCTCT |  |  |  |
| *β-actin*-F | ACCACCATGATGCAGAAGGA | 60 | 154 | 100 |
| *β-actin*-R | CCTGCTTGCTGATCCACATC |  |  |  |

**Table S3. Basic information for the RNA sequencing data and mapping.**

| Sample name | Art-1 | Art-2 | Art-3 | MD-1 | MD-2 | MD-3 |
| --- | --- | --- | --- | --- | --- | --- |
| Raw reads (Million） | 42.66 | 42.01 | 40.80 | 45.09 | 41.09 | 43.44 |
| Clean reads (Million） | 42.21 | 41.62 | 40.12 | 44.77 | 40.67 | 43.01 |
| UMI reads (Million） | 40.64 | 40.02 | 38.54 | 43.02 | 38.20 | 40.30 |
| Raw bases (Gb) | 6.40 | 6.30 | 6.12 | 6.76 | 6.16 | 6.52 |
| Clean bases (Gb) | 6.33 | 6.24 | 6.02 | 6.72 | 6.10 | 6.45 |
| Error rate (%) | 0.03 | 0.03 | 0.03 | 0.03 | 0.03 | 0.03 |
| Q20 (%) | 97.69 | 97.70 | 97.71 | 97.50 | 97.78 | 97.71 |
| Q30 (%) | 93.46 | 93.54 | 93.47 | 92.95 | 93.53 | 93.47 |
| GC content (%) | 49.93 | 49.92 | 49.93 | 49.20 | 50.10 | 50.12 |
| UMI2Clean (%) | 96.26 | 96.16 | 96.05 | 96.08 | 93.93 | 93.69 |
| Dedup2Mapped UMI (%) | 66.19 | 67.37 | 68.08 | 65.71 | 67.81 | 67.56 |
| Deduped ratio (%) | 33.81 | 32.63 | 31.92 | 34.29 | 32.19 | 32.44 |
| UMI reads (Million） | 40.64 | 40.02 | 38.54 | 43.02 | 38.20 | 40.30 |
| UMI mapped (%) | 96.87 | 96.79 | 97.01 | 96.79 | 96.66 | 96.98 |
| Multiple mapped (%) | 7.57 | 7.48 | 7.26 | 7.20 | 8.21 | 6.57 |
| Uniquely mapped (%) | 89.30 | 89.31 | 89.74 | 89.59 | 88.45 | 90.41 |
| Read-1 (%) | 44.88 | 44.92 | 45.11 | 45.25 | 44.40 | 45.49 |
| Read-2 (%) | 44.42 | 44.40 | 44.63 | 44.33 | 44.05 | 44.92 |
| Reads map to '+' (%) | 44.63 | 44.62 | 44.86 | 44.80 | 44.22 | 45.19 |
| Reads map to '-' (%) | 44.67 | 44.70 | 44.88 | 44.79 | 44.23 | 45.22 |
| Nonsplice reads (%) | 39.23 | 39.65 | 39.50 | 39.39 | 38.00 | 40.20 |
| Splice reads (%) | 50.07 | 49.66 | 50.24 | 50.20 | 50.45 | 50.21 |

**Table S4. All DEGs list.**

| **Description** | **Gene_name** | **Readcount_Art** | **Readcount_MD** | **Log2FoldChange** | ***P*_adj** |
| --- | --- | --- | --- | --- | --- |
| Alpha-2-macroglobulin-like protein 1 | *a2ml1* | 9999.14 | 2783.99 | 1.84 | 2.10E-02 |
| Acetoacetyl-CoA synthetase | *aacs* | 20.50 | 350.32 | -4.09 | 2.81E-11 |
| L-aminoadipate-semialdehyde dehydrogenase-phosphopantetheinyl transferase | *aasdhppt* | 17.80 | 47.74 | -1.42 | 4.19E-02 |
| Alpha-aminoadipic semialdehyde synthase, mitochondrial | *aass* | 110.19 | 347.52 | -1.66 | 8.77E-05 |
| Protein AATF | *aatf* | 14.90 | 41.40 | -1.47 | 4.06E-02 |
| Bile salt export pump | *abcb11* | 2374.53 | 7176.48 | -1.60 | 9.87E-08 |
| ATP-binding cassette sub-family B member 6, mitochondrial | *abcb6* | 229.36 | 469.85 | -1.03 | 6.38E-03 |
| Canalicular multispecific organic anion transporter 1 | *abcc2* | 1227.11 | 3080.55 | -1.33 | 2.86E-05 |
| Multidrug resistance-associated protein 4 | *abcc4* | 127.44 | 338.20 | -1.41 | 9.02E-05 |
| ATP-binding cassette sub-family D member 2 | *abcd2* | 31.72 | 80.68 | -1.35 | 3.49E-02 |
| ATP-binding cassette sub-family E member 1 | *abce1* | 316.06 | 679.80 | -1.10 | 1.97E-03 |
| ATP-binding cassette sub-family F member 1 | *abcf1* | 185.93 | 406.70 | -1.13 | 2.98E-03 |
| ATP-binding cassette sub-family F member 2 | *abcf2* | 42.12 | 287.88 | -2.77 | 1.14E-14 |
| ATP-binding cassette sub-family F member 3 | *abcf3* | 29.34 | 123.70 | -2.08 | 3.88E-04 |
| ATP-binding cassette sub-family G member 2 | *abcg2* | 310.66 | 86.18 | 1.85 | 5.42E-08 |
| Mycophenolic acid acyl-glucuronide esterase, mitochondrial | *abhd10* | 173.50 | 63.90 | 1.44 | 2.88E-04 |
| Monoacylglycerol lipase ABHD12 | *abhd12* | 828.84 | 476.54 | 0.80 | 4.10E-02 |
| Protein ABHD15 | *abhd15* | 48.22 | 8.48 | 2.51 | 6.61E-05 |
| Monoacylglycerol lipase ABHD2-A | *abhd2a* | 51.83 | 127.71 | -1.30 | 4.00E-03 |
| Acetyl-CoA carboxylase | *acaca* | 116.89 | 2424.06 | -4.37 | 6.00E-03 |
| Acyl-CoA dehydrogenase family member 11 | *acad11* | 1734.61 | 951.51 | 0.87 | 1.81E-02 |
| Very long-chain specific acyl-CoA dehydrogenase, mitochondrial | *acadl* | 2142.70 | 1116.47 | 0.94 | 6.80E-03 |
| Medium-chain specific acyl-CoA dehydrogenase, mitochondrial | *acadm* | 3238.61 | 1811.75 | 0.84 | 2.10E-02 |
| Acetyl-CoA acetyltransferase, cytosolic | *acat2* | 75.11 | 294.26 | -1.97 | 5.11E-05 |
| ATP-citrate synthase | *acly* | 46.29 | 101.96 | -1.14 | 3.09E-02 |
| Aconitate hydratase, mitochondrial | *aco2* | 1398.90 | 380.35 | 1.88 | 1.21E-04 |
| Acyl-coenzyme A thioesterase 1 | *acot1* | 32.33 | 10.45 | 1.63 | 4.69E-02 |
| Acyl-coenzyme A thioesterase 11 | *acot11* | 638.57 | 311.80 | 1.03 | 3.69E-03 |
| Long-chain-fatty-acid--CoA ligase ACSBG2 | *acsbg2* | 217.96 | 894.54 | -2.04 | 1.54E-04 |
| Long-chain-fatty-acid--CoA ligase 3 | *acsl3* | 36.42 | 97.19 | -1.42 | 1.62E-02 |
| Long-chain-fatty-acid--CoA ligase 4 | *acsl4* | 18.63 | 113.84 | -2.61 | 1.96E-03 |
| Long-chain-fatty-acid--CoA ligase 6 | *acsl6* | 160.54 | 74.33 | 1.11 | 2.10E-02 |
| Uncharacterized aarF domain-containing protein kinase 1 | *adck1* | 3.11 | 19.82 | -2.67 | 3.35E-02 |
| Atypical kinase COQ8B, mitochondrial | *adck4* | 65.57 | 209.19 | -1.67 | 1.37E-05 |
| Hydroxyacid-oxoacid transhydrogenase, mitochondrial | *adhfe1* | 894.91 | 492.34 | 0.86 | 2.18E-02 |
| Adiponectin receptor protein 2 | *adipor2* | 339.84 | 1150.54 | -1.76 | 1.53E-02 |
| ADM2 | *adm2* | 149.77 | 68.04 | 1.14 | 1.38E-02 |
| Adenylosuccinate lyase | *adsl* | 651.77 | 1144.92 | -0.81 | 4.12E-02 |
| Adenylosuccinate synthetase isozyme 1 C | *adssl1* | 30.56 | 8.65 | 1.82 | 2.85E-02 |
| Kynurenine formamidase | *afmid* | 94.57 | 437.21 | -2.21 | 2.61E-11 |
| Acylglycerol kinase, mitochondrial | *agk* | 106.40 | 204.73 | -0.94 | 4.37E-02 |
| Glycogen debranching enzyme | *agl* | 1097.17 | 565.49 | 0.96 | 1.01E-02 |
| Alkylglycerol monooxygenase | *agmo* | 2469.58 | 1239.64 | 0.99 | 2.16E-03 |
| Glycerol-3-phosphate acyltransferase 3 | *agpat9* | 236.91 | 588.23 | -1.31 | 1.62E-04 |
| Glycerol-3-phosphate acyltransferase 3-like | *agpat9l* | 72.32 | 23.80 | 1.60 | 2.72E-03 |
| Activator of 90 kDa heat shock protein ATPase homolog 1 | *ahsa1* | 51.24 | 123.05 | -1.26 | 8.02E-03 |
| Adenylate kinase 4, mitochondrial | *ak4* | 877.51 | 415.37 | 1.08 | 1.82E-03 |
| A-kinase anchor protein 9 | *akap9* | 158.97 | 64.67 | 1.30 | 2.96E-03 |
| 5-aminolevulinate synthase, nonspecific, mitochondrial | *alas1* | 6851.58 | 723.89 | 3.24 | 1.23E-02 |
| 5-aminolevulinate synthase, erythroid-specific, mitochondrial | *alas2* | 2769.16 | 801.22 | 1.79 | 4.14E-03 |
| Delta-1-pyrroline-5-carboxylate synthase | *aldh18a1* | 84.79 | 217.14 | -1.36 | 2.79E-02 |
| Retinal dehydrogenase 2 | *aldh1a2* | 278.10 | 634.19 | -1.19 | 5.61E-04 |
| Fatty aldehyde dehydrogenase | *aldh3a2* | 4054.65 | 2030.68 | 1.00 | 2.28E-03 |
| Methylmalonate-semialdehyde dehydrogenase [acylating], mitochondrial | *aldh6a1* | 6127.38 | 2863.97 | 1.10 | 4.47E-02 |
| Fructose-bisphosphate aldolase B | *aldob* | 152920.83 | 89575.36 | 0.77 | 3.40E-02 |
| S-adenosylmethionine decarboxylase proenzyme | *amd1* | 29.33 | 107.03 | -1.87 | 1.47E-04 |
| AMP deaminase 2 | *ampd2* | 325.49 | 135.46 | 1.26 | 1.12E-03 |
| Pancreatic alpha-amylase | *amy2a* | 7113.17 | 1498.26 | 2.25 | 1.17E-08 |
| Ankyrin repeat domain-containing protein 50 | *ankrd50* | 471.77 | 207.64 | 1.18 | 9.69E-04 |
| Acylamino-acid-releasing enzyme | *apeh* | 39.57 | 13.95 | 1.50 | 4.47E-02 |
| Apolipoprotein B-100 | *apob* | 15606.27 | 27851.46 | -0.84 | 2.24E-02 |
| Apolipoprotein M | *apom* | 1653.73 | 3471.15 | -1.07 | 7.02E-03 |
| MICOS complex subunit MIC26 | *apoo* | 76.22 | 234.92 | -1.62 | 1.98E-05 |
| Amyloid-beta A4 protein | *app* | 231.75 | 482.49 | -1.06 | 2.02E-02 |
| Adenine phosphoribosyltransferase | *aprt* | 21.70 | 59.75 | -1.46 | 1.54E-02 |
| Aquaporin-7 | *aqp7* | 1030.15 | 395.88 | 1.38 | 2.83E-05 |
| ADP-ribosylation factor 1 | *arf1* | 446.36 | 788.08 | -0.82 | 4.31E-02 |
| ADP-ribosylation factor GTPase-activating protein 2 | *arfgap2* | 179.34 | 527.03 | -1.56 | 4.83E-06 |
| Rho guanine nucleotide exchange factor 17 | *arhgef17* | 254.92 | 105.98 | 1.27 | 1.55E-03 |
| Rho guanine nucleotide exchange factor 18 | *arhgef18* | 116.85 | 53.80 | 1.12 | 2.52E-02 |
| Rho guanine nucleotide exchange factor 5 | *arhgef5* | 162.54 | 58.84 | 1.47 | 3.55E-04 |
| Armadillo repeat-containing protein 1 | *armc1* | 9.55 | 33.24 | -1.80 | 2.32E-02 |
| Aryl hydrocarbon receptor nuclear translocator-like protein 1 | *arntl* | 69.60 | 16.02 | 2.12 | 5.84E-05 |
| Aryl hydrocarbon receptor nuclear translocator-like protein 1 | *arntl* | 88.29 | 6.50 | 3.76 | 3.93E-13 |
| Aryl hydrocarbon receptor nuclear translocator-like protein 2 | *arntl2* | 9.63 | 34.55 | -1.84 | 1.64E-02 |
| Arrestin domain-containing protein 3 | *arrdc3* | 348.24 | 145.43 | 1.26 | 6.37E-04 |
| Ankyrin repeat and SOCS box protein 5 | *asb5* | 44.94 | 174.29 | -1.96 | 4.94E-07 |
| Aspartoacylase | *aspa* | 33.44 | 126.53 | -1.92 | 7.36E-06 |
| Aspartyl/asparaginyl beta-hydroxylase | *asph* | 129.34 | 356.01 | -1.46 | 8.59E-04 |
| ATPase family AAA domain-containing protein 3 | *atad3a* | 47.75 | 167.67 | -1.81 | 8.69E-06 |
| Bifunctional purine biosynthesis protein PURH | *atic* | 503.98 | 961.28 | -0.93 | 2.04E-02 |
| Manganese-transporting ATPase 13A1 | *atp13a1* | 72.09 | 166.10 | -1.20 | 6.49E-03 |
| Probable cation-transporting ATPase 13A3 | *atp13a3* | 972.07 | 498.24 | 0.96 | 8.80E-03 |
| V-type proton ATPase 116 kDa subunit a isoform 2 | *atp6v0a2* | 155.09 | 286.25 | -0.88 | 4.06E-02 |
| V-type proton ATPase subunit d 1 | *atp6v0d1* | 146.00 | 272.51 | -0.90 | 3.91E-02 |
| ATP synthase mitochondrial F1 complex assembly factor 1 | *atpaf1* | 83.19 | 184.47 | -1.15 | 8.60E-03 |
| Ataxin-1 | *atxn1* | 59.61 | 21.18 | 1.49 | 9.14E-03 |
| Arginine vasopressin-induced protein 1 | *avpi1* | 140.20 | 55.35 | 1.34 | 1.58E-03 |
| Antizyme inhibitor 1 | *azin1* | 1300.58 | 3101.64 | -1.25 | 7.86E-05 |
| Beta-2-microglobulin | *b2m* | 398.06 | 170.27 | 1.23 | 1.51E-02 |
| Beta-secretase 1 | *bace1* | 18.14 | 137.77 | -2.93 | 8.94E-12 |
| Gamma-butyrobetaine dioxygenase | *bbox1* | 648.21 | 316.69 | 1.03 | 3.35E-03 |
| B-cell CLL/lymphoma 9 protein | *bcl9* | 144.29 | 66.78 | 1.11 | 3.89E-02 |
| Class E basic helix-loop-helix protein 40 | *bhlhe40* | 865.50 | 226.59 | 1.93 | 4.76E-03 |
| Class E basic helix-loop-helix protein 41 | *bhlhe41* | 1.64 | 114.38 | -6.13 | 3.98E-07 |
| Bleomycin hydrolase | *blmh* | 42.60 | 103.72 | -1.28 | 1.21E-02 |
| B-cell linker protein-like | *blnk* | 136.60 | 252.87 | -0.89 | 4.47E-02 |
| Ribosome biogenesis protein BMS1 homolog | *bms1* | 44.64 | 117.61 | -1.40 | 2.56E-03 |
| Vesicle transport protein SEC20 | *bnip1* | 5.90 | 27.64 | -2.23 | 1.04E-02 |
| Ribosome biogenesis protein bop1 | *bop1* | 115.42 | 269.69 | -1.22 | 1.94E-03 |
| Valacyclovir hydrolase | *bphl* | 596.26 | 254.28 | 1.23 | 1.93E-03 |
| Ribosome biogenesis protein BRX1 homolog | *brix1* | 36.09 | 95.48 | -1.40 | 4.32E-03 |
| Peregrin | *brpf1* | 67.23 | 24.60 | 1.45 | 1.05E-02 |
| Transcription factor BTF3 homolog 4 | *btf3l4* | 142.95 | 334.31 | -1.23 | 1.43E-03 |
| Butyrophilin subfamily 1 member A1 | *btn1a1* | 46.06 | 8.28 | 2.48 | 4.03E-03 |
| butyrophilin-like protein 2 | *btnl2* | 10.02 | 39.48 | -1.98 | 5.36E-03 |
| Ester hydrolase C11orf54 homolog | *c11orf54* | 114.70 | 305.77 | -1.41 | 1.24E-04 |
| Myeloid-derived growth factor | *c19orf10* | 147.10 | 617.15 | -2.07 | 9.70E-07 |
| Specifically androgen-regulated gene protein | *c1orf116* | 238.70 | 105.25 | 1.18 | 1.93E-03 |
| Uncharacterized protein C1orf131 | *c1orf131* | 8.96 | 35.82 | -2.00 | 8.01E-03 |
| Complement component 1 Q subcomponent-binding protein, mitochondrial | *c1qbp* | 173.40 | 481.50 | -1.47 | 1.98E-05 |
| Complement C3 | *c3* | 3917.94 | 6771.48 | -0.79 | 3.49E-02 |
| TPA-induced transmembrane protein | *c3orf52* | 21.39 | 63.39 | -1.57 | 6.85E-03 |
| CAD protein | *cad* | 33.73 | 201.96 | -2.58 | 1.11E-11 |
| Calreticulin | *calr* | 147.12 | 655.87 | -2.16 | 1.02E-11 |
| Calreticulin | *calr* | 3036.64 | 8651.84 | -1.51 | 5.74E-07 |
| Calumenin-A | *calua* | 166.17 | 444.20 | -1.42 | 2.96E-03 |
| Calumenin-B | *calub* | 180.13 | 620.10 | -1.78 | 2.61E-08 |
| Soluble calcium-activated nucleotidase 1 | *cant1* | 73.11 | 164.34 | -1.17 | 7.56E-03 |
| Calnexin | *canx* | 830.62 | 2087.48 | -1.33 | 4.10E-05 |
| Calpain-2 catalytic subunit | *capn2* | 0.35 | 9.75 | -4.80 | 2.13E-02 |
| Catalase | *cat* | 8774.82 | 3871.20 | 1.18 | 1.31E-04 |
| Carbonyl reductase [NADPH] 1 | *cbr1* | 2187.44 | 1065.24 | 1.04 | 1.50E-03 |
| Cystathionine beta-synthase | *cbsl* | 1838.19 | 9209.90 | -2.32 | 1.56E-15 |
| E3 SUMO-protein ligase CBX4 | *cbx4* | 111.12 | 310.10 | -1.48 | 3.99E-02 |
| Chromobox protein homolog 7 | *cbx7* | 49.95 | 15.06 | 1.73 | 9.28E-03 |
| Coiled-coil domain-containing protein 134 | *ccdc134* | 30.31 | 81.82 | -1.43 | 6.80E-03 |
| Coiled-coil domain-containing protein 43 | *ccdc43* | 9.53 | 33.65 | -1.82 | 2.27E-02 |
| Coiled-coil domain-containing protein 86 | *ccdc86* | 37.84 | 115.81 | -1.61 | 3.75E-04 |
| Protein Daple | *ccdc88c* | 113.32 | 32.54 | 1.80 | 7.14E-05 |
| C-C motif chemokine 25 | *ccl25* | 191.25 | 77.83 | 1.30 | 2.21E-02 |
| Cyclin-G2 | *ccng2* | 205.91 | 76.25 | 1.43 | 2.42E-04 |
| Nocturnin | *ccrn4l* | 120.96 | 28.13 | 2.10 | 1.55E-04 |
| Serine-rich coiled-coil domain-containing protein 2 | *ccser2* | 30.38 | 114.40 | -1.91 | 8.39E-04 |
| CD151 antigen | *cd151* | 33.41 | 79.43 | -1.25 | 2.66E-02 |
| Macrosialin | *cd68* | 2077.35 | 942.52 | 1.14 | 7.14E-04 |
| CD81 antigen | *cd81* | 265.79 | 144.79 | 0.88 | 4.01E-02 |
| CD97 antigen | *cd97* | 292.88 | 103.64 | 1.50 | 3.94E-05 |
| Cyclin-dependent kinase-like 5 | *cdkl5* | 359.47 | 188.34 | 0.93 | 2.10E-02 |
| Cyclin-dependent kinase inhibitor 1B | *cdkn1b* | 113.28 | 29.13 | 1.96 | 1.07E-02 |
| Protein CDV3 homolog | *cdv3* | 55.62 | 120.05 | -1.11 | 2.16E-02 |
| Centromere protein V | *cenpv* | 58.45 | 23.18 | 1.33 | 2.80E-02 |
| Centrosomal protein of 192 kDa | *cep192* | 104.93 | 42.29 | 1.31 | 8.18E-03 |
| Centrosomal protein of 78 kDa | *cep78* | 45.16 | 12.07 | 1.90 | 4.32E-03 |
| Centrosomal protein of 85 kDa | *cep85* | 48.06 | 122.60 | -1.35 | 2.56E-03 |
| Ceramide synthase 1 | *cers1* | 278.51 | 44.07 | 2.66 | 4.08E-06 |
| complement factor I | *cfi* | 8542.41 | 23923.21 | -1.49 | 6.89E-07 |
| Glutathione-specific gamma-glutamylcyclotransferase 1 | *chac1* | 120.12 | 297.42 | -1.31 | 8.45E-03 |
| Chondroadherin | *chad* | 98.72 | 30.64 | 1.69 | 1.22E-03 |
| Coiled-coil-helix-coiled-coil-helix domain-containing protein 10, mitochondrial | *chchd10* | 128.32 | 256.55 | -1.00 | 2.27E-02 |
| Coiled-coil-helix-coiled-coil-helix domain-containing protein 2 | *chchd2* | 140.43 | 322.62 | -1.20 | 1.78E-03 |
| Mitochondrial intermembrane space import and assembly protein 40 | *chchd4* | 9.91 | 35.99 | -1.86 | 1.41E-02 |
| Chromodomain-helicase-DNA-binding protein 3 | *chd3* | 126.90 | 59.99 | 1.08 | 3.74E-02 |
| Chromodomain-helicase-DNA-binding protein 4 | *chd4* | 293.53 | 529.37 | -0.85 | 3.61E-02 |
| Circadian-associated transcriptional repressor | *ciart* | 2.81 | 60.31 | -4.43 | 1.32E-11 |
| Circadian-associated transcriptional repressor | *ciart* | 21.57 | 405.36 | -4.23 | 3.56E-21 |
| Circadian-associated transcriptional repressor | *ciart* | 11.29 | 83.07 | -2.88 | 1.67E-06 |
| Protein capicua homolog | *cic* | 131.69 | 64.47 | 1.03 | 4.35E-02 |
| CLOCK-interacting pacemaker | *cipc* | 72.99 | 861.51 | -3.56 | 2.84E-13 |
| CDGSH iron-sulfur domain-containing protein 3, mitochondrial | *cisd3* | 48.38 | 128.52 | -1.41 | 1.61E-03 |
| Cip1-interacting zinc finger protein | *ciz1* | 128.40 | 61.94 | 1.05 | 3.45E-02 |
| Cytoskeleton-associated protein 4 | *ckap4* | 4.43 | 25.91 | -2.55 | 4.20E-03 |
| Circadian locomoter output cycles protein kaput | *clock* | 91.58 | 28.23 | 1.70 | 3.88E-04 |
| Circadian locomoter output cycles protein kaput | *clock* | 21.74 | 1.44 | 3.91 | 2.82E-04 |
| Cleft lip and palate transmembrane protein 1 homolog | *clptm1* | 77.28 | 220.79 | -1.51 | 8.51E-05 |
| Cleft lip and palate transmembrane protein 1-like protein | *clptm1l* | 80.22 | 242.30 | -1.59 | 3.14E-05 |
| ATP-dependent Clp protease ATP-binding subunit clpX-like, mitochondrial | *clpx* | 633.50 | 361.72 | 0.81 | 4.37E-02 |
| Calsyntenin-1 | *clstn1* | 9.18 | 48.29 | -2.40 | 6.74E-03 |
| Clustered mitochondria protein homolog | *cluh* | 89.41 | 283.55 | -1.67 | 1.50E-03 |
| UMP-CMP kinase | *cmpk1* | 24.35 | 91.19 | -1.90 | 1.12E-03 |
| Protein canopy-1 | *cnpy1* | 179.79 | 501.09 | -1.48 | 1.29E-05 |
| Collagen alpha-1(XIV) chain | *col14a1* | 0.33 | 8.55 | -4.71 | 4.10E-02 |
| Collagen alpha-1(XVII) chain | *col17a1* | 79.96 | 155.91 | -0.96 | 4.09E-02 |
| Collagen alpha-2(IV) chain | *col4a2* | 13.98 | 44.15 | -1.66 | 1.77E-02 |
| Coronin-1C | *coro1c* | 115.01 | 238.33 | -1.05 | 1.41E-02 |
| Protoheme IX farnesyltransferase, mitochondrial | *cox10* | 35.46 | 83.62 | -1.24 | 2.80E-02 |
| Cytochrome c oxidase assembly protein COX15 homolog | *cox15* | 87.22 | 186.57 | -1.10 | 1.55E-02 |
| Cytochrome c oxidase subunit 4 isoform 2, mitochondrial | *cox4i2* | 1580.75 | 321.95 | 2.30 | 7.94E-05 |
| Cytochrome c oxidase subunit 5B, mitochondrial | *cox5b* | 156.36 | 373.31 | -1.26 | 8.73E-04 |
| Carboxypeptidase N subunit 2 | *cpn2* | 66.71 | 278.49 | -2.06 | 7.70E-03 |
| Copine-3 | *cpne3* | 56.57 | 20.19 | 1.49 | 2.26E-02 |
| Carnitine O-acetyltransferase | *crat* | 1062.69 | 586.48 | 0.86 | 2.28E-02 |
| Carnitine O-acetyltransferase | *crat* | 410.91 | 135.88 | 1.60 | 3.36E-03 |
| Cysteine-rich with EGF-like domain protein 2 | *creld2* | 46.92 | 391.32 | -3.06 | 3.89E-17 |
| Cardiolipin synthase (CMP-forming) | *crls1* | 81.66 | 217.68 | -1.41 | 3.21E-03 |
| Peroxisomal carnitine O-octanoyltransferase | *crot* | 2996.22 | 1469.88 | 1.03 | 1.89E-03 |
| Cryptochrome-1 | *cry1* | 27.56 | 110.15 | -2.00 | 1.94E-02 |
| Cryptochrome-1 | *cry1* | 544.93 | 201.98 | 1.43 | 3.43E-05 |
| Cryptochrome DASH | *cry-dash* | 90.24 | 445.77 | -2.30 | 3.04E-12 |
| Cold shock domain-containing protein E1 | *csde1* | 8226.99 | 4330.67 | 0.93 | 8.73E-03 |
| Cysteine and glycine-rich protein 1 | *csrp1* | 122.29 | 15.97 | 2.94 | 9.37E-03 |
| CTD small phosphatase-like protein 2-A | *ctdspl2* | 47.19 | 174.78 | -1.89 | 2.13E-06 |
| Cystathionine gamma-lyase | *cth* | 3027.13 | 5884.08 | -0.96 | 7.16E-03 |
| CTP synthase 1 | *ctps1* | 26.91 | 155.39 | -2.53 | 3.74E-10 |
| CTP synthase 1 | *ctps1* | 53.68 | 170.33 | -1.67 | 3.01E-05 |
| Cathepsin L1 | *ctsl* | 4240.65 | 10422.02 | -1.30 | 3.20E-05 |
| Gap junction Cx32.2 protein | *cx32.2* | 1067.57 | 2150.63 | -1.01 | 2.96E-03 |
| Coxsackievirus and adenovirus receptor homolog | *cxadr* | 288.25 | 141.94 | 1.02 | 1.68E-02 |
| Cytochrome b5 | *cyb5b* | 6456.86 | 3278.02 | 0.98 | 2.85E-02 |
| Cytochrome P450 26B1 | *cyp26b1* | 72.55 | 28.40 | 1.35 | 1.37E-02 |
| Cytochrome P450 27C1 | *cyp27c1* | 15.00 | 274.76 | -4.20 | 6.75E-04 |
| Cytochrome P450 2C70 | *cyp2c70* | 6486.36 | 2082.42 | 1.64 | 3.55E-08 |
| Cytochrome P450 2D28 | *cyp2d28a* | 188.41 | 391.31 | -1.05 | 6.35E-03 |
| Cytochrome P450 2J6 | *cyp2j6* | 1937.99 | 951.74 | 1.03 | 2.55E-02 |
| Cytochrome P450 2J6 | *cyp2j6* | 2377.07 | 1037.32 | 1.20 | 6.66E-03 |
| cytochrome P450 2K1-like | *cyp2k1* | 946.61 | 127.97 | 2.89 | 3.38E-20 |
| Cytochrome P450 4B1 | *cyp4b1* | 514.62 | 175.25 | 1.55 | 3.89E-06 |
| Lanosterol 14-alpha demethylase | *cyp51a1* | 82.18 | 976.55 | -3.57 | 2.37E-07 |
| Death-associated protein-like 1-A | *dapl1* | 310.70 | 115.47 | 1.43 | 7.71E-05 |
| DCN1-like protein 4 | *dcun1d4* | 85.56 | 39.38 | 1.12 | 3.49E-02 |
| N(G),N(G)-dimethylarginine dimethylaminohydrolase 1 | *ddah1* | 41.53 | 113.39 | -1.45 | 2.17E-03 |
| Probable ATP-dependent RNA helicase DDX17 | *ddx17* | 891.94 | 320.54 | 1.48 | 5.11E-06 |
| ATP-dependent RNA helicase DDX24 | *ddx24* | 63.72 | 145.91 | -1.20 | 8.38E-03 |
| Probable ATP-dependent RNA helicase DDX5 | *ddx5* | 140.17 | 812.19 | -2.53 | 6.72E-16 |
| ATP-dependent RNA helicase DDX54 | *ddx54* | 96.14 | 245.28 | -1.35 | 4.40E-04 |
| Probable ATP-dependent RNA helicase DDX56 | *ddx56* | 52.53 | 115.43 | -1.14 | 2.21E-02 |
| Differentially expressed in FDCP 6 homolog | *def6* | 904.68 | 487.93 | 0.89 | 1.62E-02 |
| DENN domain-containing protein 3 | *dennd3* | 45.37 | 14.60 | 1.64 | 1.41E-02 |
| Derlin-1 | *derl1* | 245.21 | 460.09 | -0.91 | 2.26E-02 |
| Diacylglycerol kinase kappa | *dgkk* | 28.63 | 79.64 | -1.48 | 5.66E-03 |
| Delta(24)-sterol reductase | *dhcr24* | 287.17 | 1082.41 | -1.91 | 5.19E-10 |
| Dehydrogenase/reductase SDR family member 13 | *dhrs13* | 254.63 | 695.27 | -1.45 | 1.02E-02 |
| dehydrogenase/reductase SDR family member 13-like | *dhrs13* | 21.02 | 2.39 | 3.14 | 4.54E-03 |
| Pre-mRNA-splicing factor ATP-dependent RNA helicase DHX15 | *dhx15* | 130.77 | 274.71 | -1.07 | 8.01E-03 |
| Disrupted in renal carcinoma protein 2 homolog | *dirc2* | 33.84 | 134.15 | -1.99 | 1.08E-04 |
| Disrupted in schizophrenia 1 protein | *disc1* | 171.40 | 74.61 | 1.20 | 4.00E-03 |
| H/ACA ribonucleoprotein complex subunit 4 | *dkc1* | 635.30 | 1151.98 | -0.86 | 2.49E-02 |
| Dimethylglycine dehydrogenase, mitochondrial | *dmgdh* | 12003.60 | 2755.71 | 2.12 | 1.10E-06 |
| DnaJ homolog subfamily A member 2 | *dnaja2* | 286.54 | 561.70 | -0.97 | 1.09E-02 |
| DnaJ homolog subfamily A member 3, mitochondrial | *dnaja3* | 10.87 | 35.89 | -1.72 | 3.10E-02 |
| DnaJ homolog subfamily B member 11 | *dnajb11* | 244.82 | 740.58 | -1.60 | 7.18E-07 |
| DnaJ homolog subfamily B member 4 | *dnajb4* | 38.89 | 9.82 | 1.99 | 4.76E-03 |
| DnaJ homolog subfamily B member 6 | *dnajb6* | 9.30 | 36.73 | -1.98 | 7.13E-03 |
| DnaJ homolog subfamily B member 9 | *dnajb9* | 103.86 | 232.18 | -1.16 | 4.19E-03 |
| DnaJ homolog subfamily C member 11 | *dnajc11* | 124.43 | 250.15 | -1.01 | 1.86E-02 |
| DnaJ homolog subfamily C member 3 | *dnajc3* | 199.49 | 774.08 | -1.96 | 1.82E-07 |
| DnaJ homolog subfamily C member 4 | *dnajc4* | 69.67 | 144.98 | -1.06 | 2.44E-02 |
| Deoxyhypusine hydroxylase | *dohh* | 134.32 | 312.71 | -1.22 | 3.21E-02 |
| Protein Dok-7 | *dok7* | 18.75 | 63.27 | -1.75 | 1.94E-03 |
| Protein dopey-2 | *dopey2* | 124.92 | 320.02 | -1.36 | 6.25E-03 |
| Dolichol-phosphate mannosyltransferase subunit 1 | *dpm1* | 142.93 | 286.82 | -1.00 | 1.80E-02 |
| Dipeptidyl peptidase 3 | *dpp3* | 43.37 | 120.23 | -1.47 | 1.82E-03 |
| E3 ubiquitin-protein ligase DTX3L | *dtx3l* | 48.34 | 18.67 | 1.37 | 4.17E-02 |
| E3 ubiquitin-protein ligase DTX3L | *dtx3l* | 104.59 | 35.18 | 1.57 | 4.95E-03 |
| tRNA-dihydrouridine(20) synthase [NAD(P)+]-like | *dus2* | 19.32 | 67.72 | -1.81 | 1.00E-03 |
| Dual specificity protein phosphatase 7 | *dusp7* | 181.15 | 89.66 | 1.01 | 2.66E-02 |
| Cytoplasmic dynein 1 heavy chain 1 | *dync1h1* | 701.44 | 1595.28 | -1.19 | 2.91E-04 |
| Dual specificity tyrosine-phosphorylation-regulated kinase 2 | *dyrk2* | 109.68 | 212.88 | -0.96 | 3.09E-02 |
| Probable glutamate--tRNA ligase, mitochondrial | *ears2* | 21.18 | 58.83 | -1.47 | 1.80E-02 |
| 3-beta-hydroxysteroid-Delta(8),Delta(7)-isomerase | *ebp* | 35.59 | 229.20 | -2.69 | 6.45E-08 |
| Extracellular matrix protein 1 | *ecm1* | 433.34 | 767.65 | -0.82 | 3.54E-02 |
| Endothelial differentiation-related factor 1 homolog | *edf1* | 624.32 | 1129.37 | -0.86 | 2.70E-02 |
| Elongation factor 2 | *eef2* | 6.42 | 597.54 | -6.54 | 1.31E-02 |
| Eukaryotic elongation factor 2 kinase | *eef2k* | 192.51 | 49.75 | 1.95 | 3.36E-07 |
| Interferon-induced, double-stranded RNA-activated protein kinase | *eif2ak2* | 117.89 | 51.64 | 1.19 | 1.47E-02 |
| Eukaryotic translation initiation factor 4E | *eif4e* | 349.79 | 742.94 | -1.09 | 2.82E-03 |
| Eukaryotic translation initiation factor 4E type 2 | *eif4e2* | 136.29 | 64.91 | 1.07 | 2.19E-02 |
| Eukaryotic translation initiation factor 4E type 3 | *eif4e3* | 269.83 | 127.81 | 1.08 | 2.89E-02 |
| Eukaryotic translation initiation factor 4E-binding protein 3-like | *eif4ebp3l* | 570.22 | 1554.15 | -1.45 | 5.54E-06 |
| Eukaryotic translation initiation factor 4 gamma 1 | *eif4g1* | 432.35 | 1209.11 | -1.48 | 3.12E-06 |
| Eukaryotic translation initiation factor 6 | *eif6* | 295.68 | 572.38 | -0.95 | 1.52E-02 |
| ELM2 and SANT domain-containing protein 1 | *elmsan1* | 142.78 | 67.80 | 1.07 | 2.31E-02 |
| Elongation of very long chain fatty acids protein 4 | *elovl4* | 334.99 | 593.39 | -0.82 | 4.33E-02 |
| Elongation of very long chain fatty acids protein 5 | *elovl5* | 111.17 | 526.96 | -2.24 | 6.39E-12 |
| Elongation of very long chain fatty acids protein 5 | *elovl5* | 508.06 | 936.24 | -0.88 | 1.95E-02 |
| Endonuclease G, mitochondrial | *endog* | 21.47 | 78.50 | -1.87 | 4.21E-03 |
| Ectonucleoside triphosphate diphosphohydrolase 5 | *entpd5* | 32.86 | 85.69 | -1.38 | 7.11E-03 |
| Ectonucleoside triphosphate diphosphohydrolase 7 | *entpd7* | 26.24 | 80.58 | -1.62 | 1.66E-03 |
| Band 4.1-like protein 5 | *epb41l5* | 404.22 | 914.63 | -1.18 | 4.34E-04 |
| Epoxide hydrolase 1 | *ephx1* | 164.06 | 385.19 | -1.23 | 6.75E-04 |
| Epidermal growth factor receptor kinase substrate 8-like protein 1 | *eps8l1* | 79.03 | 33.83 | 1.22 | 2.84E-02 |
| Receptor tyrosine-protein kinase erbB-2 | *erbb2* | 324.45 | 148.63 | 1.13 | 3.26E-03 |
| Serine/threonine-protein kinase/endoribonuclease IRE1 | *ern1* | 113.02 | 243.54 | -1.11 | 2.30E-02 |
| ERBB receptor feedback inhibitor 1 | *errfi1* | 787.85 | 2992.06 | -1.93 | 3.59E-02 |
| ESF1 homolog | *esf1* | 85.55 | 166.14 | -0.96 | 4.84E-02 |
| Electron transfer flavoprotein-ubiquinone oxidoreductase, mitochondrial | *etfdh* | 1147.92 | 555.14 | 1.05 | 2.23E-03 |
| Persulfide dioxygenase ETHE1, mitochondrial | *ethe1* | 139.59 | 286.63 | -1.04 | 1.25E-02 |
| Exosome complex component RRP40 | *exosc3* | 33.51 | 84.92 | -1.34 | 1.27E-02 |
| Proteinase-activated receptor 1 | *f2r* | 190.73 | 93.12 | 1.03 | 1.73E-02 |
| Fatty acid-binding protein, liver-type | *fabp1* | 14496.54 | 6872.35 | 1.08 | 5.91E-04 |
| Fatty acid desaturase 2 | *fads2* | 6.11 | 935.96 | -7.26 | 4.45E-04 |
| Protein FAM173A | *fam173a* | 94.92 | 24.59 | 1.95 | 2.14E-03 |
| Protein FAM222B | *fam222b* | 14.34 | 48.54 | -1.76 | 6.26E-03 |
| Mitoguardin 1 | *fam73a* | 294.17 | 571.65 | -0.96 | 1.47E-02 |
| Fatty acid synthase | *fasn* | 107.22 | 1850.41 | -4.11 | 4.00E-02 |
| Fas-activated serine/threonine kinase | *fastk* | 95.30 | 227.67 | -1.26 | 1.89E-03 |
| Fas-binding factor 1 homolog | *fbf1* | 119.25 | 41.70 | 1.52 | 6.59E-04 |
| rRNA 2'-O-methyltransferase fibrillarin | *fbl* | 118.37 | 512.41 | -2.11 | 8.19E-11 |
| Fructose-1,6-bisphosphatase 1 | *fbp1* | 16537.36 | 8771.09 | 0.91 | 2.10E-02 |
| F-box/LRR-repeat protein 3 | *fbxl3* | 57.49 | 7.29 | 2.98 | 3.41E-04 |
| F-box only protein 48 | *fbxo48* | 11.63 | 68.46 | -2.56 | 1.67E-06 |
| FCH and double SH3 domains protein 2 | *fchsd2* | 518.37 | 211.45 | 1.29 | 9.90E-04 |
| Farnesyl pyrophosphate synthase | *fdps* | 95.20 | 323.94 | -1.77 | 2.85E-02 |
| Fibroblast growth factor 19 | *fgf19* | 33.53 | 1.93 | 4.12 | 4.31E-02 |
| Bis(5'-adenosyl)-triphosphatase | *fhit* | 85.31 | 19.46 | 2.13 | 1.29E-05 |
| Peptidyl-prolyl cis-trans isomerase FKBP1B | *fkbp1b* | 242.58 | 69.25 | 1.81 | 3.74E-05 |
| Peptidyl-prolyl cis-trans isomerase FKBP2 | *fkbp2* | 299.66 | 691.42 | -1.21 | 2.01E-03 |
| Leucine-rich repeat transmembrane protein FLRT2 | *flrt2* | 65.06 | 17.47 | 1.90 | 7.21E-04 |
| vascular endothelial growth factor receptor 3 | *flt4* | 528.72 | 296.49 | 0.83 | 4.19E-02 |
| Ketosamine-3-kinase | *fn3krp* | 83.50 | 37.42 | 1.16 | 3.29E-02 |
| Fos-related antigen 2 | *fosl2* | 17.78 | 50.60 | -1.51 | 1.81E-02 |
| Forkhead box protein K1 | *foxk1* | 384.32 | 677.49 | -0.82 | 4.28E-02 |
| Forkhead box protein | *foxo6* | 221.13 | 448.73 | -1.02 | 3.22E-02 |
| FERM domain-containing protein 5 | *frmd5* | 122.31 | 5.47 | 4.48 | 1.21E-02 |
| Formimidoyltransferase-cyclodeaminase | *ftcd* | 7637.97 | 4424.87 | 0.79 | 3.09E-02 |
| Ferritin, heavy subunit | *fth1* | 33529.17 | 18533.29 | 0.86 | 1.65E-02 |
| Ferritin, lower subunit | *ftl* | 357.32 | 190.54 | 0.91 | 2.43E-02 |
| Frizzled-8 | *fzd8* | 88.22 | 27.40 | 1.69 | 3.76E-02 |
| Ras GTPase-activating protein-binding protein 1 | *g3bp1* | 22.62 | 59.76 | -1.40 | 2.44E-02 |
| Gamma-aminobutyric acid receptor subunit alpha-3 | *gabra3* | 24.14 | 1.26 | 4.26 | 1.03E-04 |
| UDP-glucose 4-epimerase | *gale* | 39.79 | 87.36 | -1.13 | 4.01E-02 |
| Neutral alpha-glucosidase AB | *ganab* | 303.62 | 985.14 | -1.70 | 5.38E-08 |
| H/ACA ribonucleoprotein complex subunit 1 | *gar1* | 111.81 | 296.02 | -1.40 | 1.48E-04 |
| Trifunctional purine biosynthetic protein adenosine-3 | *gart* | 76.60 | 209.28 | -1.45 | 4.95E-03 |
| Growth arrest-specific protein 2 | *gas2* | 32.75 | 540.35 | -4.04 | 5.68E-21 |
| Growth arrest-specific protein 6 | *gas6* | 409.53 | 166.58 | 1.30 | 1.94E-04 |
| 1,4-alpha-glucan-branching enzyme | *gbe1* | 11742.38 | 6692.60 | 0.81 | 2.78E-02 |
| GTP cyclohydrolase 1 | *gch1* | 1312.81 | 3730.09 | -1.51 | 9.70E-07 |
| Elongation factor G, mitochondrial | *gfm1* | 22.76 | 129.09 | -2.50 | 2.02E-08 |
| Vitamin K-dependent gamma-carboxylase | *ggcx* | 1686.85 | 2982.26 | -0.82 | 2.84E-02 |
| Glucose-induced degradation protein 4 homolog | *gid4* | 88.00 | 21.94 | 2.00 | 2.17E-05 |
| GTPase IMAP family member 8 | *gimap8* | 20.78 | 3.40 | 2.61 | 7.45E-03 |
| Ammonium transporter Rh type B-A | *gk16605* | 510.56 | 2406.75 | -2.24 | 7.45E-09 |
| Beta-galactosidase | *glb1* | 104.62 | 208.74 | -1.00 | 2.17E-02 |
| Glutaminase kidney isoform, mitochondrial | *gls* | 666.16 | 129.94 | 2.36 | 1.53E-08 |
| glutamate dehydrogenase, mitochondrial-like | *glud1* | 358.40 | 1246.01 | -1.80 | 3.88E-09 |
| Glutamate dehydrogenase, mitochondrial | *glud1* | 37127.67 | 17872.82 | 1.05 | 9.28E-04 |
| Glutamine synthetase | *glul* | 59.53 | 679.14 | -3.51 | 1.11E-14 |
| Glutamine synthetase | *glul* | 1419.68 | 7839.74 | -2.47 | 2.03E-15 |
| Guanine nucleotide-binding protein G(q) subunit alpha | *gnaq* | 116.59 | 50.42 | 1.21 | 1.05E-02 |
| Guanine nucleotide-binding protein-like 3 | *gnl3* | 97.72 | 247.70 | -1.34 | 5.17E-04 |
| Aspartate aminotransferase, cytoplasmic | *got1* | 219.65 | 443.15 | -1.01 | 1.03E-02 |
| Glycerol-3-phosphate acyltransferase 1, mitochondrial | *gpam* | 273.62 | 862.99 | -1.66 | 1.43E-07 |
| G patch domain-containing protein 4 | *gpatch4* | 21.20 | 52.54 | -1.31 | 4.18E-02 |
| Glycerophosphocholine phosphodiesterase GPCPD1 | *gpcpd1* | 941.39 | 1973.52 | -1.07 | 3.76E-02 |
| Integral membrane protein GPR137B | *gpr137b* | 105.02 | 18.55 | 2.50 | 5.71E-07 |
| G-protein coupled receptor family C group 6 member A | *gprc6a* | 1.37 | 12.18 | -3.15 | 4.25E-02 |
| Alanine aminotransferase 2 | *gpt2* | 309.83 | 109.44 | 1.50 | 1.86E-05 |
| GrpE protein homolog 1, mitochondrial | *grpel1* | 155.48 | 345.50 | -1.15 | 2.66E-03 |
| Eukaryotic peptide chain release factor GTP-binding subunit ERF3A | *gspt1* | 276.59 | 508.78 | -0.88 | 3.14E-02 |
| Glutathione S-transferase A4 | *gsta4* | 7441.81 | 4108.38 | 0.86 | 3.47E-02 |
| Glutathione S-transferase A | *gsto1* | 4009.43 | 2292.05 | 0.81 | 3.12E-02 |
| Glycogen [starch] synthase, liver | *gys2* | 3805.54 | 2121.40 | 0.84 | 1.86E-02 |
| 2-hydroxyacyl-CoA lyase 1 | *hacl1* | 394.43 | 200.33 | 0.98 | 8.94E-03 |
| Hydroxyacyl-coenzyme A dehydrogenase, mitochondrial | *hadh* | 4448.38 | 2026.68 | 1.13 | 3.82E-04 |
| Hydroxyacid oxidase 1 | *hao1* | 893.32 | 3394.51 | -1.93 | 7.63E-05 |
| HEAT repeat-containing protein 3 | *heatr3* | 52.21 | 200.78 | -1.94 | 3.22E-07 |
| Heme-binding protein 1 | *hebp1* | 220.56 | 80.44 | 1.46 | 1.39E-04 |
| Headcase protein homolog | *heca* | 83.29 | 36.35 | 1.20 | 2.58E-02 |
| Helicase with zinc finger domain 2 | *helz2* | 247.43 | 74.49 | 1.73 | 6.59E-04 |
| Homocysteine-responsive endoplasmic reticulum-resident ubiquitin-like domain member 1 protein | *herpud1* | 75.46 | 176.90 | -1.23 | 3.36E-03 |
| Hippocampus abundant transcript-like protein 1 | *hiatl1* | 50.47 | 104.05 | -1.04 | 4.17E-02 |
| Hypoxia-inducible factor 1-alpha | *hif1a* | 286.14 | 764.78 | -1.42 | 1.21E-03 |
| HIG1 domain family member 1A, mitochondrial | *higd1a* | 110.59 | 300.56 | -1.44 | 2.21E-02 |
| Homeodomain-interacting protein kinase 1 | *hipk1* | 42.20 | 4.08 | 3.37 | 2.24E-03 |
| Histone H1.5 | *hist1h1b* | 835.13 | 353.12 | 1.24 | 4.04E-04 |
| Hepatic leukemia factor | *hlf* | 68.95 | 357.79 | -2.38 | 8.30E-05 |
| Hepatic leukemia factor | *hlf* | 145.31 | 412.71 | -1.51 | 1.15E-05 |
| High mobility group protein B2 | *hmgb2* | 446.90 | 228.39 | 0.97 | 1.39E-02 |
| 3-hydroxy-3-methylglutaryl-coenzyme A reductase | *hmgcr* | 223.31 | 551.14 | -1.30 | 1.54E-04 |
| 3-hydroxy-3-methylglutaryl-coenzyme A reductase | *hmgcr* | 444.97 | 1083.49 | -1.28 | 7.57E-05 |
| Hydroxymethylglutaryl-CoA synthase, cytoplasmic | *hmgcs1* | 870.00 | 2022.97 | -1.22 | 5.38E-03 |
| Heterogeneous nuclear ribonucleoprotein A/B | *hnrnpab* | 310.48 | 729.00 | -1.23 | 3.39E-04 |
| Heterogeneous nuclear ribonucleoprotein L | *hnrnpl* | 69.28 | 154.43 | -1.16 | 3.82E-02 |
| Haptoglobin | *hp* | 929.05 | 3760.72 | -2.02 | 1.24E-06 |
| Heterochromatin protein 1-binding protein 3 | *hp1bp3* | 73.40 | 26.82 | 1.45 | 8.03E-03 |
| 2-iminobutanoate/2-iminopropanoate deaminase | *hrsp12* | 2478.67 | 1126.80 | 1.14 | 4.03E-03 |
| 17-beta-hydroxysteroid dehydrogenase 14 | *hsd17b14* | 65.30 | 147.43 | -1.17 | 1.07E-02 |
| Endoplasmin | *hsp90b1* | 5212.54 | 17084.46 | -1.71 | 1.09E-04 |
| Heat shock 70 kDa protein 4 | *hspa4* | 55.13 | 157.15 | -1.51 | 3.10E-04 |
| Endoplasmic reticulum chaperone BiP | *hspa5* | 842.17 | 3069.33 | -1.87 | 3.74E-10 |
| Stress-70 protein, mitochondrial | *hspa9* | 538.38 | 1004.73 | -0.90 | 1.77E-02 |
| 60 kDa heat shock protein, mitochondrial | *hspd1* | 408.25 | 772.35 | -0.92 | 3.06E-02 |
| Hydrocephalus-inducing protein | *hydin* | 4.61 | 121.82 | -4.73 | 3.53E-02 |
| Hypoxia up-regulated protein 1 | *hyou1* | 220.92 | 1548.33 | -2.81 | 1.85E-05 |
| Isoleucine--tRNA ligase, cytoplasmic | *iars* | 124.18 | 280.20 | -1.17 | 2.03E-02 |
| Isopentenyl-diphosphate Delta-isomerase 1 | *idi1* | 170.55 | 703.25 | -2.04 | 2.59E-07 |
| Interferon-induced helicase C domain-containing protein 1 | *ifih1* | 137.89 | 61.38 | 1.17 | 1.60E-02 |
| Interferon-related developmental regulator 1 | *ifrd1* | 363.71 | 923.61 | -1.34 | 3.88E-04 |
| Interferon-related developmental regulator 1 | *ifrd1* | 72.78 | 168.63 | -1.21 | 4.95E-03 |
| Intraflagellar transport protein 80 homolog | *ift80* | 121.35 | 49.04 | 1.31 | 5.03E-03 |
| Insulin-like growth factor-binding protein complex acid labile subunit | *igfals* | 2631.11 | 6040.23 | -1.20 | 2.19E-04 |
| Immunoglobulin gamma-1 heavy chain | *igg1* | 271.13 | 113.01 | 1.26 | 1.27E-03 |
| Immunoglobulin superfamily member 8 | *igsf8* | 155.90 | 78.53 | 0.99 | 4.65E-02 |
| Protein turtle homolog A | *igsf9* | 248.51 | 130.49 | 0.93 | 4.56E-02 |
| Interleukin-6 receptor subunit beta | *il6st* | 274.78 | 29.47 | 3.22 | 9.87E-04 |
| Inhibitor of growth protein 2 | *ing2* | 36.59 | 10.53 | 1.80 | 1.10E-02 |
| Inhibin beta B chain | *inhbb* | 4.08 | 24.22 | -2.57 | 3.49E-02 |
| Insulin-induced gene 1 protein | *insig1* | 1268.92 | 565.25 | 1.17 | 6.33E-04 |
| Importin-4 | *ipo4* | 49.48 | 168.72 | -1.77 | 1.02E-05 |
| Interferon regulatory factor 6 | *irf6* | 547.71 | 248.28 | 1.14 | 5.43E-03 |
| Inositol-3-phosphate synthase 1-A | *isyna1* | 12.42 | 39.63 | -1.67 | 1.92E-02 |
| Inositol-trisphosphate 3-kinase A | *itpka* | 137.40 | 41.34 | 1.73 | 1.52E-02 |
| Protein Jumonji | *jarid2* | 139.83 | 67.41 | 1.05 | 2.44E-02 |
| Junction-mediating and -regulatory protein | *jmy* | 18.33 | 100.01 | -2.45 | 1.46E-02 |
| Kalirin | *kalrn* | 196.55 | 101.25 | 0.96 | 3.08E-02 |
| Histone acetyltransferase KAT2B | *kat2b* | 187.45 | 66.60 | 1.49 | 1.24E-04 |
| Potassium channel subfamily K member 13 | *kcnk13* | 164.42 | 79.18 | 1.05 | 2.02E-02 |
| Potassium channel subfamily K member 5 | *kcnk5* | 240.88 | 78.96 | 1.61 | 1.37E-05 |
| ER lumen protein-retaining receptor 2 | *kdelr2* | 87.70 | 228.96 | -1.38 | 4.01E-02 |
| Pumilio homolog 3 | *kiaa0020* | 68.99 | 162.18 | -1.23 | 4.45E-03 |
| Uncharacterized protein KIAA0232 | *kiaa0232* | 227.99 | 112.47 | 1.02 | 2.17E-02 |
| Sushi domain-containing protein 6 | *kiaa0247* | 16.37 | 44.10 | -1.43 | 4.80E-02 |
| Sushi domain-containing protein 6 | *kiaa0247* | 170.78 | 404.05 | -1.24 | 5.38E-03 |
| Uncharacterized protein KIAA0895-like | *kiaa0895l* | 5.87 | 25.54 | -2.12 | 2.10E-02 |
| Putative monooxygenase P33monox | *kiaa1191* | 1202.71 | 346.56 | 1.80 | 2.76E-03 |
| Centrosomal protein kizuna | *kiz* | 84.04 | 37.15 | 1.18 | 3.51E-02 |
| Krueppel-like factor 15 | *klf15* | 6.89 | 68.61 | -3.32 | 1.18E-02 |
| Krueppel-like factor 9 | *klf9* | 71.59 | 404.64 | -2.50 | 5.42E-08 |
| Kelch domain-containing protein 4 | *klhdc4* | 20.11 | 91.65 | -2.19 | 4.69E-06 |
| Histone-lysine N-methyltransferase 2A | *kmt2a* | 124.41 | 59.30 | 1.07 | 4.35E-02 |
| Importin subunit alpha-1 | *kpna2* | 66.07 | 373.24 | -2.50 | 2.84E-13 |
| Kinase suppressor of Ras 1 | *ksr1* | 102.80 | 15.43 | 2.74 | 4.23E-09 |
| Cytosol aminopeptidase | *lap3* | 159.17 | 336.88 | -1.08 | 6.25E-03 |
| La-related protein 4 | *larp4* | 73.68 | 227.17 | -1.62 | 1.37E-05 |
| Leucine--tRNA ligase, cytoplasmic | *lars* | 144.37 | 424.57 | -1.56 | 6.76E-06 |
| Prolyl 3-hydroxylase 3 | *leprel2* | 25.40 | 120.19 | -2.24 | 3.00E-02 |
| Galectin-9B | *lgals9b* | 1257.27 | 381.07 | 1.72 | 1.68E-08 |
| LMBR1 domain-containing protein 2-B | *lmbrd2b* | 317.50 | 762.68 | -1.26 | 5.04E-03 |
| UPF0687 protein C20orf27 homolog | *loc562078* | 113.54 | 51.59 | 1.14 | 2.38E-02 |
| Lon protease homolog, mitochondrial | *lonp1* | 151.18 | 366.76 | -1.28 | 4.31E-04 |
| Lysophosphatidic acid receptor 1 | *lpar1* | 67.25 | 24.65 | 1.45 | 8.16E-03 |
| Acyl-CoA:lysophosphatidylglycerol acyltransferase 1 | *lpgat1* | 71.29 | 395.40 | -2.47 | 2.58E-02 |
| Phosphatidate phosphatase LPIN1 | *lpin1* | 157.95 | 2725.27 | -4.11 | 6.61E-03 |
| Leucine-rich alpha-2-glycoprotein | *lrg1* | 463.79 | 3364.93 | -2.86 | 6.95E-22 |
| Alpha-2-macroglobulin receptor-associated protein | *lrpap1* | 60.74 | 178.77 | -1.56 | 1.18E-03 |
| Leucine-rich PPR motif-containing protein, mitochondrial | *lrpprc* | 127.54 | 340.07 | -1.41 | 1.10E-04 |
| Lanosterol synthase | *lss* | 24.19 | 76.56 | -1.66 | 1.86E-03 |
| Leukotriene A-4 hydrolase | *lta4h* | 793.67 | 1903.89 | -1.26 | 1.00E-04 |
| Latent-transforming growth factor beta-binding protein 3 | *ltbp3* | 92.05 | 226.49 | -1.30 | 9.13E-04 |
| Cilia- and flagella-associated protein 91 | *maats1* | 100.54 | 44.57 | 1.17 | 2.27E-02 |
| Protein mago nashi homolog | *magoh* | 100.71 | 258.81 | -1.36 | 4.16E-02 |
| Mucosa-associated lymphoid tissue lymphoma translocation protein 1 | *malt1* | 203.39 | 410.18 | -1.01 | 7.38E-03 |
| Mesencephalic astrocyte-derived neurotrophic factor | *manf* | 117.45 | 350.75 | -1.58 | 5.29E-06 |
| Amine oxidase [flavin-containing] A | *maoa* | 504.85 | 115.81 | 2.12 | 3.05E-11 |
| Amine oxidase [flavin-containing] | *maoa* | 2139.31 | 393.14 | 2.44 | 6.68E-05 |
| Microtubule-associated protein 1A | *map1a* | 186.30 | 93.65 | 0.99 | 2.53E-02 |
| MAP7 domain-containing protein 1 | *map7d1* | 278.94 | 803.87 | -1.53 | 5.91E-03 |
| Methionine--tRNA ligase, cytoplasmic | *mars* | 21.26 | 163.20 | -2.94 | 1.02E-12 |
| S-adenosylmethionine synthase isoform type-2 | *mat2a* | 45.25 | 197.90 | -2.13 | 1.18E-04 |
| Protein MB21D2 | *mb21d2* | 41.94 | 14.67 | 1.52 | 3.68E-02 |
| Midasin | *mdn1* | 154.15 | 396.47 | -1.36 | 4.78E-03 |
| NADP-dependent malic enzyme, mitochondrial | *me3* | 62.75 | 275.11 | -2.13 | 4.08E-07 |
| LRP chaperone MESD | *mesdc2* | 80.08 | 169.12 | -1.08 | 1.60E-02 |
| Mesoderm-specific transcript homolog protein | *mest* | 279.66 | 145.46 | 0.94 | 3.49E-02 |
| Methyltransferase-like protein 13 | *mettl13* | 15.98 | 53.30 | -1.74 | 5.16E-03 |
| Microfibril-associated glycoprotein 4 | *mfap4* | 32.27 | 196.56 | -2.61 | 2.15E-07 |
| Protein O-GlcNAcase | *mgea5* | 57.10 | 181.86 | -1.67 | 3.14E-05 |
| Protein O-GlcNAcase | *mgea5* | 249.54 | 90.98 | 1.46 | 1.39E-04 |
| Monoglyceride lipase | *mgll* | 529.75 | 44.12 | 3.59 | 1.43E-26 |
| Mid1-interacting protein 1-B | *mid1ip1b* | 0.65 | 13.84 | -4.40 | 1.65E-02 |
| Mid1-interacting protein 1-like | *mid1ip1b* | 38.45 | 139.45 | -1.86 | 7.69E-05 |
| Mannosyl-oligosaccharide glucosidase | *mogs* | 113.70 | 307.59 | -1.44 | 8.01E-05 |
| Putative helicase mov-10-B.2 | *mov10b.2* | 24.46 | 4.99 | 2.29 | 1.97E-02 |
| Mitochondrial pyruvate carrier 1 | *mpc1* | 2113.31 | 1023.96 | 1.05 | 1.94E-03 |
| 55 kDa erythrocyte membrane protein | *mpp1* | 186.79 | 361.07 | -0.95 | 1.81E-02 |
| Protein Mpv17 | *mpv17* | 294.75 | 125.67 | 1.23 | 8.87E-04 |
| Major histocompatibility complex class I-related gene protein | *mr1* | 200.09 | 82.62 | 1.28 | 1.09E-02 |
| 39S ribosomal protein L2, mitochondrial | *mrpl37* | 93.25 | 186.86 | -1.00 | 3.09E-02 |
| 39S ribosomal protein L9, mitochondrial | *mrpl9* | 42.34 | 96.62 | -1.19 | 2.72E-02 |
| 28S ribosomal protein S21, mitochondrial | *mrps21* | 56.68 | 142.49 | -1.33 | 2.56E-03 |
| 28S ribosomal protein S30, mitochondrial | *mrps30* | 38.45 | 101.92 | -1.41 | 4.03E-03 |
| 28S ribosomal protein S28, mitochondrial | *mrps35* | 69.21 | 161.82 | -1.23 | 5.78E-03 |
| Methylsterol monooxygenase 1 | *msmo1* | 3100.21 | 7804.13 | -1.33 | 3.39E-04 |
| Methionine-R-sulfoxide reductase B1-A | *msrb1a* | 82.95 | 20.28 | 2.03 | 4.51E-04 |
| Methionine-R-sulfoxide reductase B2, mitochondrial | *msrb2* | 736.98 | 351.97 | 1.07 | 2.30E-03 |
| NADP-dependent mannitol dehydrogenase | *mtd* | 1.03 | 48.62 | -5.57 | 9.13E-09 |
| Protein LYRIC | *mtdh* | 347.93 | 678.58 | -0.96 | 1.02E-02 |
| Transcription termination factor 2, mitochondrial | *mterf2* | 4.42 | 21.98 | -2.31 | 2.43E-02 |
| Myotubularin-related protein 4 | *mtmr4* | 186.27 | 63.75 | 1.55 | 1.00E-04 |
| Protein MTO1 homolog, mitochondrial | *mto1* | 8.32 | 36.62 | -2.14 | 3.54E-03 |
| Mevalonate kinase | *mvk* | 36.58 | 83.95 | -1.20 | 2.79E-02 |
| Myb-binding protein 1A-like protein | *mybbp1a* | 233.32 | 431.84 | -0.89 | 3.26E-02 |
| Myocardial zonula adherens protein | *myzap* | 182.76 | 81.87 | 1.16 | 5.92E-03 |
| Neuroblastoma-amplified sequence | *nbas* | 99.93 | 197.16 | -0.98 | 2.83E-02 |
| Nucleolin | *ncl* | 10.76 | 86.54 | -3.01 | 1.92E-09 |
| Nucleolin | *ncl* | 1482.67 | 2920.55 | -0.98 | 8.38E-03 |
| Nicalin-1 | *ncl1* | 54.72 | 166.47 | -1.61 | 8.14E-05 |
| Protein NDRG1 | *ndrg1* | 2964.41 | 1028.08 | 1.53 | 5.41E-04 |
| Negative elongation factor A | *nelfa* | 886.50 | 505.01 | 0.81 | 4.35E-02 |
| Nuclear factor interleukin-3-regulated protein | *nfil3* | 54.37 | 331.51 | -2.61 | 3.45E-05 |
| Nuclear factor interleukin-3-regulated protein | *nfil3* | 75.80 | 15.58 | 2.28 | 5.86E-06 |
| Nuclear factor interleukin-3-regulated protein | *nfil3* | 29.77 | 3.65 | 3.03 | 6.48E-04 |
| Nuclear factor NF-kappa-B p100 subunit | *nfkb2* | 223.30 | 412.05 | -0.88 | 2.78E-02 |
| NF-X1-type zinc finger protein NFXL1 | *nfxl1* | 47.52 | 102.21 | -1.11 | 3.84E-02 |
| neugrin-like | *ngrn* | 96.27 | 25.10 | 1.94 | 2.16E-03 |
| H/ACA ribonucleoprotein complex subunit 2-like protein | *nhp2* | 93.05 | 230.84 | -1.31 | 8.43E-04 |
| MKI67 FHA domain-interacting nucleolar phosphoprotein | *nifk* | 151.65 | 319.21 | -1.07 | 6.65E-03 |
| Magnesium transporter NIPA4 | *nipal4* | 4.36 | 146.78 | -5.07 | 8.65E-05 |
| protein NLRC3-like | *nlrc3* | 219.01 | 111.89 | 0.97 | 3.10E-02 |
| Protein NLRC3 | *nlrc3* | 94.23 | 36.14 | 1.38 | 8.51E-03 |
| NLR family CARD domain-containing protein 3 | *nlrc3* | 47.35 | 17.35 | 1.45 | 4.35E-02 |
| NLR family CARD domain-containing protein 3 | *nlrc3* | 218.48 | 75.51 | 1.53 | 1.69E-04 |
| NACHT, LRR and PYD domains-containing protein 3 | *nlrp3* | 523.76 | 237.19 | 1.14 | 8.60E-03 |
| 60S ribosomal export protein NMD3 | *nmd3* | 2011.28 | 1168.62 | 0.78 | 4.00E-02 |
| Nucleoside diphosphate kinase 3 | *nme3* | 36.56 | 131.58 | -1.85 | 1.84E-05 |
| Nucleoside diphosphate kinase, mitochondrial | *nme4* | 2806.09 | 600.81 | 2.22 | 7.14E-14 |
| Nicotinamide riboside kinase 2 | *nmrk2* | 4358.31 | 1711.83 | 1.35 | 3.44E-05 |
| Nucleolar complex protein 2 homolog | *noc2l* | 70.11 | 157.31 | -1.17 | 9.63E-03 |
| Nucleolar protein 6 | *nol6* | 33.06 | 76.00 | -1.20 | 4.22E-02 |
| Nucleolar and coiled-body phosphoprotein 1 | *nolc1* | 37.18 | 100.90 | -1.44 | 2.60E-03 |
| H/ACA ribonucleoprotein complex subunit 3 | *nop10* | 68.68 | 313.20 | -2.19 | 3.19E-10 |
| Nucleolar protein 10 | *nop10* | 27.24 | 77.94 | -1.52 | 4.13E-03 |
| Nucleolar protein 58 | *nop58* | 113.73 | 266.03 | -1.23 | 1.46E-03 |
| Neuronal PAS domain-containing protein 2 | *npas2* | 123.84 | 26.30 | 2.24 | 1.82E-07 |
| NPC intracellular cholesterol transporter 1 | *npc1* | 595.07 | 260.46 | 1.19 | 6.72E-04 |
| Nuclear receptor subfamily 0 group B member 2 | *nr0b2* | 329.65 | 75.89 | 2.12 | 4.76E-10 |
| Nuclear receptor subfamily 1 group D member 1 | *nr1d1* | 4.13 | 210.73 | -5.67 | 3.89E-17 |
| Nuclear receptor subfamily 1 group D member 1 | *nr1d1* | 1.76 | 59.90 | -5.09 | 9.47E-03 |
| Nuclear receptor subfamily 1 group D member 2 | *nr1d2* | 1.00 | 75.07 | -6.22 | 2.10E-02 |
| Nuclear receptor subfamily 2 group F member 6 | *nr2f6* | 276.14 | 117.98 | 1.23 | 1.32E-03 |
| Neuronal cell adhesion molecule | *nrcam* | 1473.44 | 823.07 | 0.84 | 2.62E-02 |
| Neuropilin-1a | *nrp1a* | 170.00 | 82.83 | 1.04 | 2.41E-02 |
| Sterol-4-alpha-carboxylate 3-dehydrogenase, decarboxylating | *nsdhl* | 55.54 | 280.71 | -2.34 | 8.95E-10 |
| NSFL1 cofactor p47 | *nsfl1c* | 219.49 | 517.08 | -1.24 | 4.96E-04 |
| NUAK family SNF1-like kinase 2 | *nuak2* | 412.87 | 191.27 | 1.11 | 1.98E-03 |
| Nucleoside diphosphate-linked moiety X motif 6 | *nudt6* | 70.01 | 157.00 | -1.17 | 1.26E-02 |
| Nuclear pore complex protein Nup93 | *nup93* | 359.44 | 79.75 | 2.17 | 9.75E-11 |
| Oral-facial-digital syndrome 1 protein homolog | *ofd1* | 27.79 | 7.79 | 1.84 | 3.95E-02 |
| 2-oxoglutarate dehydrogenase, mitochondrial | *ogdh* | 2017.16 | 1098.17 | 0.88 | 1.45E-02 |
| 2-oxoglutarate dehydrogenase-like, mitochondrial | *ogdhl* | 469.28 | 27.06 | 4.12 | 2.29E-14 |
| Oxysterol-binding protein-related protein 3 | *osbpl3* | 5.72 | 51.06 | -3.16 | 1.43E-02 |
| Oxysterol-binding protein-related protein 9 | *osbpl9* | 390.41 | 199.91 | 0.97 | 1.78E-02 |
| Oxidative stress-induced growth inhibitor 2 | *osgin2* | 162.96 | 85.32 | 0.93 | 4.28E-02 |
| Succinyl-CoA:3-ketoacid coenzyme A transferase 1, mitochondrial | *oxct1* | 2878.21 | 1682.64 | 0.77 | 3.78E-02 |
| Polyadenylate-binding protein 4 | *pabpc4* | 2124.09 | 1057.71 | 1.01 | 1.21E-02 |
| Polyadenylate-binding protein 2 | *pabpn1* | 49.63 | 104.13 | -1.07 | 3.79E-02 |
| Phosphofurin acidic cluster sorting protein 2 | *pacs2* | 68.42 | 19.06 | 1.84 | 6.49E-04 |
| Procollagen C-endopeptidase enhancer 1 | *pcolce* | 855.24 | 1640.78 | -0.94 | 8.64E-03 |
| Ethanolamine-phosphate cytidylyltransferase | *pcyt2* | 4.06 | 20.08 | -2.31 | 3.35E-02 |
| Protein RRP5 homolog | *pdcd11* | 132.22 | 320.68 | -1.28 | 2.78E-03 |
| 2',5'-phosphodiesterase 12 | *pde12* | 17.83 | 56.46 | -1.66 | 5.37E-03 |
| cAMP-specific 3',5'-cyclic phosphodiesterase 4B | *pde4b* | 81.46 | 34.65 | 1.23 | 2.72E-02 |
| Protein disulfide-isomerase A4 | *pdia4* | 1105.55 | 5099.41 | -2.21 | 1.84E-09 |
| Protein disulfide-isomerase A5 | *pdia5* | 170.71 | 327.33 | -0.94 | 1.96E-02 |
| Protein disulfide-isomerase A6 | *pdia6* | 487.13 | 1855.03 | -1.93 | 8.78E-11 |
| PDZ and LIM domain protein 4 | *pdlim4* | 212.85 | 104.32 | 1.03 | 1.36E-02 |
| Sister chromatid cohesion protein PDS5 homolog B | *pds5b* | 200.68 | 100.28 | 1.00 | 2.10E-02 |
| Decaprenyl-diphosphate synthase subunit 1 | *pdss1* | 9.15 | 29.88 | -1.71 | 4.99E-02 |
| Phosphatidylethanolamine N-methyltransferase | *pemt* | 596.47 | 167.95 | 1.83 | 1.47E-08 |
| Xaa-Pro dipeptidase | *pepd* | 188.18 | 431.22 | -1.20 | 1.56E-02 |
| Alpha-aspartyl dipeptidase | *pepe* | 690.01 | 325.34 | 1.08 | 1.93E-03 |
| Period circadian protein homolog 1 | *per1* | 288.22 | 866.58 | -1.59 | 7.18E-07 |
| Pescadillo homolog | *pes1* | 217.77 | 545.97 | -1.33 | 1.24E-04 |
| fructose-2,6-bisphosphatase 4 | *pfkfb4* | 223.50 | 45.64 | 2.29 | 6.64E-10 |
| 6-phosphogluconate dehydrogenase, decarboxylating | *pgd* | 245.38 | 442.12 | -0.85 | 4.15E-02 |
| Glycerol-3-phosphate phosphatase | *pgp* | 13.29 | 76.96 | -2.53 | 1.16E-06 |
| Membrane-associated progesterone receptor component 1 | *pgrmc1* | 1733.75 | 980.77 | 0.82 | 2.32E-02 |
| Prohibitin | *phb* | 272.84 | 713.44 | -1.39 | 4.01E-05 |
| Polyhomeotic-like protein 1 | *phc1* | 52.48 | 18.98 | 1.47 | 1.92E-02 |
| Deoxyribodipyrimidine photo-lyase | *phr* | 6.13 | 56.35 | -3.20 | 2.20E-07 |
| Phosphatidylinositol 3-kinase regulatory subunit beta | *pik3r2* | 69.85 | 25.97 | 1.43 | 1.21E-02 |
| Phosphatidylserine decarboxylase proenzyme, mitochondrial | *pisd* | 571.04 | 1239.64 | -1.12 | 7.47E-04 |
| Phosphatidylinositol transfer protein beta isoform | *pitpnb* | 15.63 | 44.66 | -1.51 | 2.40E-02 |
| Presequence protease, mitochondrial | *pitrm1* | 59.15 | 165.64 | -1.49 | 3.36E-04 |
| Group 3 secretory phospholipase A2 | *pla2g3* | 220.31 | 856.43 | -1.96 | 2.15E-02 |
| Platelet-activating factor acetylhydrolase | *pla2g7* | 115.78 | 306.31 | -1.40 | 1.33E-04 |
| PLAC8-like protein 1 | *plac8l1* | 120.61 | 25.27 | 2.26 | 4.06E-03 |
| 1-phosphatidylinositol 4,5-bisphosphate phosphodiesterase epsilon-1 | *plce1* | 151.94 | 55.72 | 1.45 | 7.24E-04 |
| 1-phosphatidylinositol 4,5-bisphosphate phosphodiesterase gamma-1 | *plcg1* | 620.21 | 331.44 | 0.90 | 1.81E-02 |
| Plectin | *plec* | 361.55 | 142.39 | 1.34 | 2.58E-04 |
| Pleckstrin homology domain-containing family A member 6 | *plekhg6* | 701.77 | 1567.52 | -1.16 | 3.06E-02 |
| Phospholipid phosphatase 1 | *plpp1* | 29.80 | 89.93 | -1.59 | 9.81E-04 |
| Plexin-A2 | *plxna2* | 59.21 | 21.71 | 1.45 | 2.38E-02 |
| Phosphomannomutase 2 | *pmm2* | 28.86 | 83.07 | -1.53 | 3.89E-03 |
| Mitochondrial-processing peptidase subunit alpha | *pmpca* | 175.41 | 375.85 | -1.10 | 4.20E-03 |
| Phosphomevalonate kinase | *pmvk* | 36.28 | 84.26 | -1.22 | 2.83E-02 |
| Patatin-like phospholipase domain-containing protein 2 | *pnpla2* | 621.98 | 2694.48 | -2.12 | 5.00E-08 |
| Patatin-like phospholipase domain-containing protein 2 | *pnpla2* | 617.88 | 310.05 | 0.99 | 7.22E-03 |
| DNA-directed RNA polymerase I subunit RPA1 | *polr1a* | 84.57 | 181.94 | -1.11 | 9.59E-03 |
| DNA-directed RNA polymerase I subunit RPA2 | *polr1b* | 33.91 | 80.36 | -1.24 | 2.13E-02 |
| Periostin | *postn* | 22.83 | 65.67 | -1.52 | 9.54E-03 |
| Phospholipid phosphatase 3 | *ppap2b* | 246.44 | 74.31 | 1.73 | 1.73E-06 |
| Amidophosphoribosyltransferase | *ppat* | 59.18 | 161.96 | -1.45 | 1.93E-03 |
| phosphopantothenoylcysteine decarboxylase isoform X2 | *ppcdc* | 380.64 | 206.78 | 0.88 | 3.84E-02 |
| Periplakin | *ppl* | 100.35 | 191.87 | -0.94 | 4.47E-02 |
| protein phosphatase 1K, mitochondrial-like | *ppm1k* | 325.83 | 21.46 | 3.92 | 1.02E-05 |
| Protein phosphatase 1L | *ppm1l* | 14.08 | 49.82 | -1.82 | 3.23E-03 |
| Protein phosphatase 1 regulatory subunit 37 | *ppp1r37* | 371.27 | 205.69 | 0.85 | 4.72E-02 |
| Protein phosphatase 1 regulatory subunit 3G | *ppp1r3g* | 605.86 | 3203.09 | -2.40 | 4.62E-04 |
| Peroxisome proliferator-activated receptor gamma coactivator-related protein 1 | *pprc1* | 25.73 | 98.00 | -1.93 | 6.49E-03 |
| Peroxisome proliferator-activated receptor gamma coactivator-related protein 1 | *pprc1* | 19.88 | 63.01 | -1.66 | 4.03E-03 |
| Protein phosphatase PTC7 homolog | *pptc7* | 31.69 | 78.39 | -1.31 | 2.20E-02 |
| Lysosomal amino acid transporter 1 homolog | *pqlc2* | 100.09 | 218.66 | -1.13 | 5.78E-03 |
| Perforin-1 | *prf1* | 36.34 | 6.51 | 2.48 | 1.04E-03 |
| Perforin-1 | *prf1* | 15.27 | 1.72 | 3.15 | 3.47E-02 |
| Proteoglycan 4 | *prg4* | 1378.11 | 3252.10 | -1.24 | 8.30E-05 |
| Protein kinase C delta type | *prkcd* | 30.32 | 75.23 | -1.31 | 2.10E-02 |
| Glucosidase 2 subunit beta | *prkcsh* | 205.77 | 459.69 | -1.16 | 1.33E-03 |
| Vitamin K-dependent protein S | *pros1* | 4635.17 | 2631.86 | 0.82 | 2.84E-02 |
| Exopolyphosphatase PRUNE1 | *prune* | 130.53 | 296.93 | -1.19 | 3.92E-02 |
| Protein prune homolog 2 | *prune2* | 75.23 | 21.79 | 1.79 | 1.93E-02 |
| Proteasome subunit alpha type-4 | *psma4* | 65.19 | 141.80 | -1.12 | 1.75E-02 |
| Proteasome subunit beta type-7 | *psmb7* | 155.90 | 288.48 | -0.89 | 4.89E-02 |
| 26S proteasome regulatory subunit 7 | *psmc2* | 125.92 | 278.61 | -1.15 | 4.64E-03 |
| 26S proteasome non-ATPase regulatory subunit 11A | *psmd11a* | 40.13 | 101.27 | -1.34 | 6.94E-03 |
| 26S proteasome non-ATPase regulatory subunit 2 | *psmd2* | 331.10 | 629.15 | -0.93 | 1.53E-02 |
| 26S proteasome non-ATPase regulatory subunit 3 | *psmd3* | 331.56 | 639.18 | -0.95 | 1.47E-02 |
| 26S proteasome non-ATPase regulatory subunit 8 | *psmd8* | 120.71 | 233.84 | -0.95 | 3.37E-02 |
| Prostaglandin E synthase 3 | *ptges3* | 37.56 | 85.73 | -1.19 | 3.25E-02 |
| Receptor-type tyrosine-protein phosphatase N2 | *ptprn2* | 5.54 | 35.88 | -2.70 | 2.31E-03 |
| Periodic tryptophan protein 2 homolog | *pwp2* | 46.75 | 186.14 | -1.99 | 2.13E-07 |
| Peroxidasin | *pxdn* | 187.88 | 72.76 | 1.37 | 4.14E-03 |
| Pregnancy zone protein | *pzp* | 236.96 | 68.89 | 1.78 | 6.82E-03 |
| Nicotinate-nucleotide pyrophosphorylase [carboxylating] (Fragment) | *qprt* | 432.65 | 872.47 | -1.01 | 4.33E-03 |
| Ras-related protein Rab-20 | *rab20* | 362.86 | 170.75 | 1.09 | 4.17E-03 |
| Ras-related protein Rab-33B | *rab33b* | 0.36 | 13.25 | -5.20 | 2.51E-03 |
| Ras-related protein Rab-39A | *rab39a* | 96.30 | 24.13 | 2.00 | 1.22E-05 |
| Ras-like protein family member 11A-like | *rasl11a* | 484.63 | 261.46 | 0.89 | 2.10E-02 |
| RNA-binding protein 38 | *rbm38* | 62.90 | 25.52 | 1.30 | 4.41E-02 |
| RNA-binding protein 47 | *rbm47* | 133.35 | 61.46 | 1.12 | 1.92E-02 |
| retinol-binding protein 2 | *rbp2* | 813.69 | 418.13 | 0.96 | 4.32E-02 |
| Retinol dehydrogenase 12 | *rdh12* | 29.35 | 140.67 | -2.26 | 9.98E-05 |
| Retinol dehydrogenase 12 | *rdh12* | 3088.50 | 1732.63 | 0.83 | 2.61E-02 |
| Retinol dehydrogenase 8 | *rdh8* | 1077.99 | 574.82 | 0.91 | 1.09E-02 |
| Transcription factor p65 | *rela* | 225.96 | 410.02 | -0.86 | 4.75E-02 |
| GTP-binding protein REM 1 | *rem1* | 7.25 | 52.36 | -2.85 | 4.64E-02 |
| Proto-oncogene tyrosine-protein kinase receptor Ret | *ret* | 491.92 | 1906.98 | -1.95 | 1.15E-07 |
| Raftlin | *rftn1* | 19.77 | 0.64 | 4.95 | 5.94E-05 |
| Ral guanine nucleotide dissociation stimulator-like 1 | *rgl1* | 144.13 | 69.50 | 1.05 | 2.82E-02 |
| RPE-retinal G protein-coupled receptor | *rgr* | 178.40 | 57.88 | 1.62 | 2.49E-05 |
| Ammonium transporter Rh type A | *rhag* | 310.28 | 96.02 | 1.69 | 3.86E-02 |
| Rho-related BTB domain-containing protein 2 | *rhobtb2* | 683.40 | 293.85 | 1.22 | 3.34E-04 |
| Ribonuclease H2 subunit B | *rnaseh2b* | 40.62 | 12.77 | 1.67 | 1.93E-02 |
| Probable E3 ubiquitin-protein ligase RNF144A-A | *rnf144aa* | 66.40 | 29.15 | 1.19 | 4.75E-02 |
| RING finger protein 223 | *rnf223* | 6.23 | 67.53 | -3.44 | 1.20E-02 |
| Nuclear receptor ROR-beta | *rorb* | 230.68 | 71.85 | 1.68 | 5.12E-06 |
| Nuclear receptor ROR-beta | *rorb* | 519.05 | 104.59 | 2.31 | 3.93E-13 |
| Ribonuclease P protein subunit p30 | *rpp30* | 4.14 | 28.00 | -2.76 | 1.33E-03 |
| Ribonuclease P protein subunit p40 | *rpp40* | 23.53 | 77.14 | -1.71 | 8.87E-04 |
| 40S ribosomal protein S27-like | *rps27l* | 61.48 | 156.30 | -1.35 | 3.68E-02 |
| 40S ribosomal protein SA | *rpsa* | 213.81 | 74.63 | 1.52 | 4.40E-03 |
| Ribosome-binding protein 1 | *rrbp1* | 75.70 | 202.33 | -1.42 | 2.30E-03 |
| Ribosome-binding protein 1 | *rrbp1* | 223.82 | 576.09 | -1.36 | 6.82E-05 |
| RRP12-like protein | *rrp12* | 39.00 | 233.88 | -2.58 | 3.04E-12 |
| Ribosome biogenesis regulatory protein homolog | *rrs1* | 74.88 | 167.94 | -1.17 | 7.16E-03 |
| tRNA-splicing ligase RtcB homolog | *rtcb* | 191.47 | 428.47 | -1.16 | 1.93E-03 |
| Protein S100-A1 | *s100a1* | 44.79 | 282.44 | -2.66 | 7.11E-03 |
| Serum amyloid A-3 protein | *saa3* | 132.71 | 29.16 | 2.19 | 3.15E-02 |
| Sacsin | *sacs* | 145.07 | 33.91 | 2.10 | 2.76E-05 |
| Sphingomyelin synthase-related protein 1 | *samd8* | 117.43 | 352.29 | -1.59 | 6.65E-06 |
| sterile alpha motif domain-containing protein 9-like | *samd9l* | 152.86 | 47.24 | 1.69 | 6.41E-04 |
| GTP-binding protein SAR1a | *sar1a* | 537.80 | 972.88 | -0.86 | 2.78E-02 |
| Spindle assembly abnormal protein 6 homolog | *sass6* | 123.36 | 50.28 | 1.29 | 3.10E-03 |
| Diamine acetyltransferase 2 | *sat2* | 20.36 | 52.16 | -1.36 | 4.47E-02 |
| DNA-binding protein SATB1 | *satb1* | 305.47 | 1.31 | 7.87 | 1.68E-08 |
| Secretory carrier-associated membrane protein 4 | *scamp4* | 54.69 | 111.73 | -1.03 | 4.71E-02 |
| S phase cyclin A-associated protein in the endoplasmic reticulum | *scaper* | 230.51 | 113.11 | 1.03 | 1.69E-02 |
| Lysosome membrane protein 2 | *scarb2* | 1915.79 | 933.11 | 1.04 | 2.42E-03 |
| Secernin-2 | *scrn2* | 9.97 | 34.59 | -1.80 | 2.02E-02 |
| Signal peptide, CUB and EGF-like domain-containing protein 2 | *scube2* | 388.54 | 190.78 | 1.03 | 1.46E-02 |
| Stromal cell-derived factor 2-like protein 1 | *sdf2l1* | 210.35 | 717.39 | -1.77 | 3.00E-08 |
| Succinate dehydrogenase [ubiquinone] flavoprotein subunit, mitochondrial | *sdha* | 444.96 | 898.06 | -1.01 | 5.70E-03 |
| Succinate dehydrogenase assembly factor 2, mitochondrial | *sdhaf2* | 158.69 | 328.65 | -1.05 | 9.89E-03 |
| Succinate dehydrogenase [ubiquinone] iron-sulfur subunit, mitochondrial | *sdhb* | 13.57 | 63.47 | -2.23 | 2.58E-04 |
| Protein SEC13 homolog | *sec13* | 108.49 | 296.61 | -1.45 | 1.60E-04 |
| Protein transport protein Sec23B | *sec23b* | 232.07 | 1131.99 | -2.29 | 4.20E-06 |
| Protein transport protein Sec24D | *sec24d* | 265.01 | 511.85 | -0.95 | 1.48E-02 |
| Protein transport protein Sec31A | *sec31a* | 37.61 | 117.45 | -1.64 | 2.23E-04 |
| Protein transport protein Sec61 subunit alpha-like 1 | *sec61a1* | 202.00 | 621.27 | -1.62 | 6.95E-03 |
| Methanethiol oxidase | *selenbp1* | 5737.84 | 2532.50 | 1.18 | 2.59E-04 |
| Semaphorin-4B | *sema4b* | 19.22 | 121.39 | -2.66 | 1.27E-04 |
| Semaphorin-6D | *sema6d* | 142.35 | 40.17 | 1.83 | 1.21E-03 |
| Selenoprotein Pa | *sepp1a* | 43256.83 | 23036.47 | 0.91 | 7.87E-03 |
| Selenoprotein Pb | *sepp1b* | 717.53 | 1370.13 | -0.93 | 8.56E-03 |
| Septin-4 | *sept4* | 149.37 | 62.07 | 1.27 | 4.53E-03 |
| Serine incorporator 5 | *serinc5* | 199.00 | 73.81 | 1.43 | 2.44E-04 |
| Alpha-1-antitrypsin homolog | *serpina1* | 214124.87 | 122881.47 | 0.80 | 2.65E-02 |
| Serpin H1 | *serpinh1* | 35.98 | 101.06 | -1.49 | 2.44E-02 |
| SERTA domain-containing protein 2 | *sertad2* | 19.75 | 102.86 | -2.38 | 5.20E-06 |
| Serine/threonine-protein kinase Sgk1 | *sgk1* | 1464.19 | 379.54 | 1.95 | 5.73E-11 |
| SHC-transforming protein 1 | *shc1* | 104.51 | 47.03 | 1.15 | 2.17E-02 |
| Probable N-acetyltransferase camello | *si:ch211-81n22.1* | 78.64 | 154.42 | -0.97 | 4.99E-02 |
| Signal-induced proliferation-associated 1-like protein 1 | *sipa1l2* | 138.73 | 31.35 | 2.15 | 8.73E-08 |
| Solute carrier family 12 member 4 | *slc12a4* | 347.81 | 188.90 | 0.88 | 3.63E-02 |
| Solute carrier family 13 member 3 | *slc13a3* | 193.64 | 1238.65 | -2.68 | 1.68E-02 |
| Solute carrier family 13 member 4 | *slc13a4* | 13.46 | 39.19 | -1.54 | 3.65E-02 |
| Monocarboxylate transporter 1 | *slc16a1* | 618.60 | 169.94 | 1.86 | 7.85E-03 |
| Monocarboxylate transporter 12-B | *slc16a12b* | 271.41 | 663.22 | -1.29 | 4.31E-02 |
| Monocarboxylate transporter 8 | *slc16a2* | 619.45 | 247.60 | 1.32 | 1.86E-04 |
| Monocarboxylate transporter 9 | *slc16a9* | 36.21 | 166.83 | -2.20 | 7.24E-04 |
| Solute carrier family 17 member 9 | *slc17a9* | 96.85 | 291.29 | -1.59 | 1.03E-05 |
| Excitatory amino acid transporter 3 | *slc1a1* | 341.01 | 185.45 | 0.88 | 3.63E-02 |
| Sodium-dependent phosphate transporter 2 | *slc20a2* | 102.25 | 279.60 | -1.45 | 6.21E-03 |
| Solute carrier family 23 member 2 | *slc23a2* | 78.34 | 191.91 | -1.29 | 3.82E-02 |
| Calcium-binding mitochondrial carrier protein SCaMC-2-A | *slc25a25* | 523.51 | 72.47 | 2.85 | 9.59E-15 |
| Solute carrier family 25 member 36-A | *slc25a36* | 121.81 | 567.62 | -2.22 | 4.14E-12 |
| Solute carrier family 25 member 39 | *slc25a39* | 342.90 | 741.62 | -1.11 | 1.26E-03 |
| Mitochondrial coenzyme A transporter SLC25A42 | *slc25a42* | 201.17 | 100.39 | 1.00 | 2.70E-02 |
| Prestin | *slc26a5* | 63.31 | 459.25 | -2.86 | 1.79E-04 |
| Long-chain fatty acid transport protein 6 | *slc27a6* | 822.14 | 1610.02 | -0.97 | 7.17E-03 |
| Equilibrative nucleoside transporter 2 | *slc29a2* | 43.44 | 110.86 | -1.35 | 4.04E-03 |
| Solute carrier family 2, facilitated glucose transporter member 8 | *slc2a8* | 241.01 | 625.05 | -1.37 | 3.88E-05 |
| Solute carrier family 2, facilitated glucose transporter member 9 | *slc2a9* | 508.18 | 255.72 | 0.99 | 9.89E-03 |
| Zinc transporter 1 | *slc30a1* | 51.23 | 150.99 | -1.56 | 1.89E-04 |
| Zinc transporter 7 | *slc30a7* | 172.51 | 344.04 | -1.00 | 3.84E-02 |
| Sodium-dependent phosphate transport protein 2A | *slc34a1* | 0.00 | 13.55 |  | 3.52E-02 |
| CMP-sialic acid transporter | *slc35a1* | 284.22 | 525.96 | -0.89 | 2.80E-02 |
| Solute carrier family 35 member B1 | *slc35b1* | 84.49 | 238.30 | -1.50 | 8.30E-05 |
| Sodium-coupled neutral amino acid transporter 2 | *slc38a2* | 77.51 | 293.04 | -1.92 | 6.49E-05 |
| Sodium-coupled neutral amino acid transporter 4 | *slc38a4* | 2184.64 | 8252.99 | -1.92 | 5.17E-11 |
| Large neutral amino acids transporter small subunit 3 | *slc43a1* | 147.39 | 272.12 | -0.88 | 4.91E-02 |
| Large neutral amino acids transporter small subunit 4 | *slc43a2* | 58.45 | 576.33 | -3.30 | 4.09E-02 |
| Multidrug and toxin extrusion protein 1 | *slc47a1* | 2102.37 | 4138.16 | -0.98 | 5.38E-03 |
| Electrogenic sodium bicarbonate cotransporter 1 | *slc4a4* | 57.58 | 13.91 | 2.05 | 4.21E-04 |
| Electroneutral sodium bicarbonate exchanger 1 | *slc4a8* | 26.48 | 6.77 | 1.97 | 2.29E-02 |
| Sodium- and chloride-dependent GABA transporter 2 | *slc6a13* | 96.47 | 16.25 | 2.57 | 6.05E-08 |
| Sodium-dependent neutral amino acid transporter B(0)AT2 | *slc6a15* | 13.43 | 48.91 | -1.86 | 1.35E-02 |
| Sodium- and chloride-dependent taurine transporter | *slc6a6* | 1495.42 | 2702.03 | -0.85 | 2.58E-02 |
| High affinity cationic amino acid transporter 1 | *slc7a1* | 21.99 | 4.73 | 2.22 | 2.40E-02 |
| Solute carrier organic anion transporter family member 1C1 | *slco1c1* | 2290.87 | 4655.08 | -1.02 | 2.62E-03 |
| SAFB-like transcription modulator | *sltm* | 241.38 | 112.14 | 1.11 | 7.80E-03 |
| Mothers against decapentaplegic homolog 1 | *smad1* | 22.86 | 76.57 | -1.74 | 9.13E-04 |
| Sphingomyelin phosphodiesterase | *smpd1* | 783.89 | 2137.14 | -1.45 | 1.55E-03 |
| Histone-lysine N-methyltransferase SMYD3 | *smyd3* | 9.63 | 34.92 | -1.86 | 1.60E-02 |
| Sterol O-acyltransferase 2 | *soat2* | 2555.43 | 900.77 | 1.50 | 2.23E-05 |
| Cell surface Cu-only superoxide dismutase 5 | *sod5* | 5.56 | 27.59 | -2.31 | 6.48E-03 |
| SPARC | *sparc* | 366.78 | 816.93 | -1.16 | 4.16E-02 |
| Spermatogenesis-associated protein 5 | *spata5* | 21.01 | 55.53 | -1.40 | 2.58E-02 |
| Msx2-interacting protein | *spen* | 501.30 | 204.13 | 1.30 | 2.19E-04 |
| SPRY domain-containing SOCS box protein 3 | *spsb3* | 59.24 | 22.83 | 1.38 | 2.10E-02 |
| SPRY domain-containing SOCS box protein 3 | *spsb3* | 84.09 | 20.13 | 2.06 | 2.44E-02 |
| Squalene monooxygenase | *sqle* | 14.47 | 248.34 | -4.10 | 1.67E-05 |
| Sulfide:quinone oxidoreductase, mitochondrial | *sqrdl* | 482.45 | 156.69 | 1.62 | 9.99E-07 |
| Sequestosome-1 | *sqstm1* | 1042.73 | 1885.70 | -0.85 | 2.02E-02 |
| Sterol regulatory element-binding protein 2 | *srebf2* | 187.58 | 434.68 | -1.21 | 1.12E-02 |
| Sterol regulatory element-binding protein 1 | *srebfp1* | 151.61 | 648.52 | -2.10 | 3.20E-04 |
| SLIT-ROBO Rho GTPase-activating protein 3 | *srgap3* | 30.39 | 7.58 | 2.00 | 2.24E-02 |
| Spermidine synthase | *srm* | 19.60 | 127.62 | -2.70 | 6.76E-10 |
| Serine/arginine-rich splicing factor 7 | *srsf7* | 41.14 | 106.01 | -1.37 | 4.63E-03 |
| Translocon-associated protein subunit alpha | *ssr1* | 410.03 | 770.01 | -0.91 | 2.96E-02 |
| Protein SSUH2 homolog | *ssuh2* | 291.38 | 541.29 | -0.89 | 2.20E-02 |
| StAR-related lipid transfer protein 7, mitochondrial | *stard7* | 72.60 | 177.16 | -1.29 | 1.61E-03 |
| StAR-related lipid transfer protein 9 | *stard9* | 101.26 | 45.67 | 1.15 | 2.28E-02 |
| Stanniocalcin-2 | *stc2* | 50.88 | 13.52 | 1.91 | 2.53E-03 |
| Serine/threonine-protein kinase 35 | *stk35* | 16.09 | 59.50 | -1.89 | 2.01E-03 |
| Sushi domain-containing protein 1 | *susd1* | 125.49 | 42.14 | 1.57 | 4.90E-04 |
| Rho GTPase-activating protein SYDE2 | *syde2* | 107.71 | 41.85 | 1.36 | 4.54E-03 |
| Heterogeneous nuclear ribonucleoprotein Q | *syncrip* | 75.25 | 211.01 | -1.49 | 1.26E-04 |
| Nesprin-2 | *syne2* | 108.92 | 14.60 | 2.90 | 3.86E-02 |
| Synaptogyrin-2 | *syngr2* | 104.50 | 197.95 | -0.92 | 4.20E-02 |
| Transforming acidic coiled-coil-containing protein 2 | *tacc2* | 230.03 | 119.14 | 0.95 | 2.70E-02 |
| TATA box-binding protein-associated factor RNA polymerase I subunit B | *taf1b* | 13.10 | 49.98 | -1.93 | 2.03E-03 |
| TATA box-binding protein-associated factor, RNA polymerase I, subunit C | *taf1c* | 67.24 | 157.14 | -1.22 | 4.68E-03 |
| talin-2 isoform X1 | *talin-2* | 50.27 | 17.13 | 1.55 | 1.77E-02 |
| Transducin beta-like protein 2 | *tbl2* | 96.14 | 206.70 | -1.10 | 1.01E-02 |
| Transcription elongation factor A protein 3 | *tcea3* | 431.78 | 236.53 | 0.87 | 2.80E-02 |
| Transcription factor 24 | *tcf24* | 112.85 | 39.83 | 1.50 | 9.44E-04 |
| Transcription factor 7-like 2 | *tcf7l2* | 48.12 | 18.23 | 1.40 | 3.99E-02 |
| Transcobalamin-2 | *tcn2* | 1203.72 | 2311.82 | -0.94 | 7.11E-03 |
| T-complex protein 11-like protein 2 | *tcp11l2* | 188.37 | 704.35 | -1.90 | 1.84E-09 |
| Methylcytosine dioxygenase TET3 | *tet3* | 144.25 | 56.37 | 1.36 | 2.87E-03 |
| Dimethyladenosine transferase 2, mitochondrial | *tfb2m* | 90.40 | 204.34 | -1.18 | 4.23E-03 |
| Transferrin receptor protein 1 | *tfrc* | 1016.89 | 550.69 | 0.88 | 2.83E-02 |
| Homeobox protein TGIF1 | *tgif1* | 61.20 | 21.60 | 1.50 | 1.02E-02 |
| Thyroid adenoma-associated protein homolog | *thada* | 87.38 | 287.72 | -1.72 | 1.67E-06 |
| THUMP domain-containing protein 3 | *thumpd3* | 114.52 | 228.44 | -1.00 | 2.12E-02 |
| Mitochondrial import inner membrane translocase subunit Tim10 | *timm10* | 68.27 | 169.86 | -1.32 | 1.92E-03 |
| Mitochondrial import inner membrane translocase subunit Tim17-A | *timm17a* | 202.44 | 368.43 | -0.86 | 3.82E-02 |
| Mitochondrial import inner membrane translocase subunit Tim23 | *timm23* | 93.03 | 185.43 | -1.00 | 3.48E-02 |
| Mitochondrial import inner membrane translocase subunit TIM44 | *timm44* | 1.04 | 23.30 | -4.49 | 4.06E-05 |
| Mitochondrial import inner membrane translocase subunit Tim8 A | *timm8a* | 87.11 | 173.60 | -0.99 | 3.76E-02 |
| Transketolase | *tkt* | 226.30 | 97.33 | 1.22 | 2.07E-03 |
| Talin-2 | *tln2* | 121.11 | 40.78 | 1.57 | 4.47E-04 |
| Toll-like receptor 5 | *tlr5* | 280.09 | 138.44 | 1.02 | 1.01E-02 |
| Transmembrane 4 L6 family member 1 | *tm4sf1* | 534.33 | 1035.91 | -0.96 | 1.63E-02 |
| Delta(14)-sterol reductase | *tm7sf2* | 217.98 | 488.96 | -1.17 | 1.57E-02 |
| Transmembrane and coiled-coil domains protein 2 | *tmcc2* | 24.36 | 364.98 | -3.91 | 1.43E-26 |
| Transmembrane emp24 domain-containing protein 9 | *tmed9* | 281.39 | 526.19 | -0.90 | 2.48E-02 |
| Transmembrane protein 165 | *tmem165* | 116.38 | 248.05 | -1.09 | 8.51E-03 |
| Transmembrane protein 198 | *tmem198* | 434.41 | 1030.71 | -1.25 | 1.39E-04 |
| Transmembrane protein 206 | *tmem206* | 584.53 | 148.60 | 1.98 | 6.78E-10 |
| Transmembrane protein 229b | *tmem229b* | 33.10 | 92.45 | -1.48 | 9.63E-03 |
| Transmembrane protein 256 | *tmem256* | 201.25 | 107.38 | 0.91 | 4.96E-02 |
| Trimeric intracellular cation channel type B | *tmem38b* | 1052.85 | 3020.70 | -1.52 | 8.21E-04 |
| Transmembrane protein 41B | *tmem41b* | 27.82 | 103.11 | -1.89 | 3.94E-05 |
| Transmembrane protein 54 | *tmem54* | 385.71 | 792.53 | -1.04 | 2.70E-02 |
| Transmembrane protein 56-B | *tmem56b* | 127.26 | 349.63 | -1.46 | 3.86E-05 |
| Sigma intracellular receptor 2 | *tmem97* | 13.63 | 84.95 | -2.64 | 1.39E-07 |
| Tumor necrosis factor ligand superfamily member 10 | *tnfsf10* | 430.13 | 205.05 | 1.07 | 8.03E-03 |
| Mitochondrial import receptor subunit T | *tomm70a* | 189.13 | 372.95 | -0.98 | 1.54E-02 |
| Torsin-1A-interacting protein 2 | *tor1aip2* | 152.72 | 367.56 | -1.27 | 3.85E-04 |
| Thymocyte selection-associated high mobility group box protein T | *tox* | 38.88 | 11.01 | 1.82 | 1.27E-02 |
| Tumor protein p53-inducible protein 11 | *tp53i11* | 211.55 | 106.00 | 1.00 | 2.04E-02 |
| Tubulin polyglutamylase complex subunit 2 | *tpgs2* | 87.14 | 35.65 | 1.29 | 1.47E-02 |
| Tryptase-2 | *tpsab1* | 111.40 | 214.09 | -0.94 | 3.76E-02 |
| Translocating chain-associated membrane protein 1-like 1 | *tram1l1* | 345.41 | 837.21 | -1.28 | 1.55E-04 |
| Tripartite motif-containing protein 16 | *trim16l* | 17.32 | 2.93 | 2.56 | 3.56E-02 |
| E3 ubiquitin-protein ligase TRIM21 | *trim21* | 36.58 | 11.24 | 1.70 | 2.40E-02 |
| E3 ubiquitin/ISG15 ligase TRIM25 | *trim25* | 321.41 | 108.75 | 1.56 | 1.37E-05 |
| Zinc-binding protein A33 | *trim35-3* | 324.37 | 105.23 | 1.62 | 3.10E-04 |
| E3 ubiquitin-protein ligase TRIM39-like | *trim39* | 299.85 | 131.11 | 1.19 | 2.18E-03 |
| Triple functional domain protein | *trio* | 68.53 | 15.10 | 2.18 | 4.12E-05 |
| Transient receptor potential cation channel subfamily M member 5 | *trpm5* | 213.21 | 107.01 | 0.99 | 2.44E-02 |
| Probable tRNA pseudouridine synthase 1 | *trub1* | 25.95 | 164.52 | -2.66 | 3.23E-11 |
| Tetraspanin-2 | *tspan2* | 292.55 | 161.16 | 0.86 | 4.89E-02 |
| Tetraspanin-8 | *tspan8* | 3377.76 | 5801.55 | -0.78 | 3.49E-02 |
| Pre-rRNA-processing protein TSR1 homolog | *tsr1* | 121.09 | 354.05 | -1.55 | 1.06E-05 |
| Tetratricopeptide repeat protein 19, mitochondrial | *ttc19* | 21.18 | 64.42 | -1.61 | 7.85E-03 |
| Tetratricopeptide repeat protein 27 | *ttc27* | 46.09 | 105.48 | -1.19 | 1.96E-02 |
| Tubulin beta-6 chain | *tubb6* | 124.35 | 51.17 | 1.28 | 7.72E-03 |
| Tumor suppressor candidate 3 | *tusc3* | 35.60 | 103.58 | -1.54 | 1.50E-03 |
| Thioredoxin domain-containing protein 11 | *txndc11* | 55.26 | 144.55 | -1.39 | 1.49E-03 |
| Ubiquitin-like modifier-activating enzyme 5 | *uba5* | 52.86 | 129.84 | -1.30 | 5.75E-03 |
| ubiquitin-like protein 3 | *ubl3* | 446.86 | 808.53 | -0.86 | 3.11E-02 |
| Nucleolar transcription factor 1-A | *ubtf* | 12.99 | 40.62 | -1.65 | 2.57E-02 |
| Uridine-cytidine kinase 1 | *uck1* | 153.12 | 47.41 | 1.69 | 5.09E-05 |
| Mitochondrial uncoupling protein 2 | *ucp2* | 42220.15 | 14998.30 | 1.49 | 3.76E-04 |
| UDP-glucose 6-dehydrogenase | *ugdh* | 1651.76 | 689.04 | 1.26 | 7.78E-05 |
| UDP-glucose:glycoprotein glucosyltransferase 1 | *uggt1* | 180.88 | 587.81 | -1.70 | 1.53E-07 |
| UDP-glucuronosyltransferase 2A1 | *ugt2a1* | 1125.38 | 512.82 | 1.13 | 7.14E-04 |
| UDP-glucuronosyltransferase 2B15 | *ugt2b15* | 517.70 | 248.75 | 1.06 | 4.20E-03 |
| Serine/threonine-protein kinase ULK1 | *ulk1* | 33.51 | 224.13 | -2.74 | 5.90E-06 |
| Serine/threonine-protein kinase ULK2 | *ulk2* | 177.72 | 735.29 | -2.05 | 2.35E-05 |
| Uricase | *uox* | 31785.16 | 16775.54 | 0.92 | 5.70E-03 |
| Cytochrome b-c1 complex subunit 1, mitochondrial | *uqcrc1* | 744.78 | 1415.24 | -0.93 | 1.18E-02 |
| Cytochrome b-c1 complex subunit 2, mitochondrial | *uqcrc2* | 346.79 | 717.61 | -1.05 | 4.54E-03 |
| Cytochrome b-c1 complex subunit Rieske, mitochondrial | *uqcrfs1* | 322.31 | 682.98 | -1.08 | 3.17E-03 |
| Cytochrome b-c1 complex subunit 6, mitochondrial | *uqcrh* | 195.14 | 430.29 | -1.14 | 2.18E-03 |
| Uroporphyrinogen-III synthase | *uros* | 128.33 | 56.19 | 1.19 | 7.80E-03 |
| Ubiquitin carboxyl-terminal hydrolase 1 | *usp1* | 68.97 | 24.83 | 1.47 | 6.66E-03 |
| U3 small nucleolar RNA-associated protein 18 homolog | *utp18* | 28.38 | 86.46 | -1.61 | 1.36E-03 |
| Small subunit processome component 20 homolog | *utp20* | 41.03 | 186.78 | -2.19 | 1.53E-08 |
| Utrophin | *utrn* | 1034.38 | 479.76 | 1.11 | 1.98E-03 |
| Vascular endothelial growth factor A | *vegfa* | 44.66 | 198.00 | -2.15 | 1.48E-03 |
| Selenoprotein S | *vimp* | 366.20 | 781.78 | -1.09 | 2.16E-03 |
| Vacuole membrane protein 1 | *vmp1* | 196.69 | 370.88 | -0.92 | 2.87E-02 |
| WW domain-binding protein 4 | *wbp4* | 198.84 | 77.33 | 1.36 | 3.48E-02 |
| Cilia- and flagella-associated protein 251 | *wdr66* | 0.35 | 45.43 | -7.02 | 4.20E-05 |
| WD repeat-containing protein 74 | *wdr74* | 29.94 | 72.73 | -1.28 | 2.72E-02 |
| X-ray repair cross-complementing protein 5 | *xrcc5* | 81.37 | 33.16 | 1.29 | 1.95E-02 |
| Tyrosine--tRNA ligase, cytoplasmic | *yars* | 24.00 | 73.12 | -1.61 | 2.79E-03 |
| ATP-dependent zinc metalloprotease YME1L1 | *yme1l1* | 293.75 | 594.03 | -1.02 | 6.49E-03 |
| Probable palmitoyltransferase ZDHHC12 | *zdhhc12* | 34.34 | 9.65 | 1.83 | 1.53E-02 |
| AN1-type zinc finger protein 5 | *zfand5* | 211.14 | 744.77 | -1.82 | 5.60E-09 |
| Zinc finger MYND domain-containing protein 19 | *zmynd19* | 11.46 | 34.52 | -1.59 | 3.98E-02 |
| Zinc finger protein 106 | *znf106* | 87.15 | 29.38 | 1.57 | 2.05E-03 |
| Zinc finger protein 292 | *znf292* | 288.12 | 144.49 | 1.00 | 1.55E-02 |
| Zinc finger protein 704 | *znf704* | 200.81 | 81.56 | 1.30 | 1.56E-03 |
| NFX1-type zinc finger-containing protein 1 | *znfx1* | 35.51 | 7.10 | 2.32 | 3.02E-02 |
| E3 ubiquitin-protein ligase znrf2 | *znrf2* | 303.75 | 81.85 | 1.89 | 4.69E-08 |
| Transcription factor VBP | *#N/A* | 17.16 | 252.97 | -3.88 | 2.58E-04 |
| Cytochrome c-b | *#N/A* | 59.43 | 444.47 | -2.90 | 7.02E-18 |
| Coenzyme Q-binding protein C | *#N/A* | 149.13 | 864.60 | -2.54 | 7.57E-08 |
| Sodium/potassium-transporting ATPase subunit beta-2 | *#N/A* | 5.53 | 21.88 | -1.98 | 4.57E-02 |
| Melanoma receptor tyrosine-protein kinase | *#N/A* | 314.41 | 1178.45 | -1.91 | 1.38E-03 |
| caspase recruitment domain-containing protein 18-like | *#N/A* | 55.52 | 202.91 | -1.87 | 1.79E-06 |
| UDP-N-acetylhexosamine pyrophosphorylase-like protein 1 isoform X4 | *#N/A* | 35.73 | 121.83 | -1.77 | 6.10E-05 |
| Multifunctional-autoprocessing repeats-in-toxin | *#N/A* | 889.89 | 2989.07 | -1.75 | 3.63E-03 |
| Uridine 5’-monophosphate synthase | *#N/A* | 55.60 | 164.67 | -1.57 | 1.17E-04 |
| UDP-glucuronosyltransferase 2B31 | *#N/A* | 486.09 | 1431.62 | -1.56 | 4.92E-07 |
| Sarcoplasmic/endoplasmic reticulum calcium ATPase 2 | *#N/A* | 123.26 | 361.02 | -1.55 | 7.14E-04 |
| Major facilitator superfamily domain-containing protein 6-B | *#N/A* | 20.30 | 58.71 | -1.53 | 9.45E-03 |
| Serine/threonine-protein kinase RI | *#N/A* | 115.02 | 326.80 | -1.51 | 3.25E-05 |
| Sarcoplasmic/endoplasmic reticulum calcium ATPase 2 | *#N/A* | 154.61 | 418.71 | -1.44 | 4.12E-05 |
| Serine/threonine-protein phosphatase 4 catalytic subunit B | *#N/A* | 67.22 | 168.05 | -1.32 | 2.03E-03 |
| SWI/SNF-related matrix-associated actin-dependent regulator of chromatin subfamily A member 5 | *#N/A* | 22.02 | 54.86 | -1.32 | 4.59E-02 |
| Cbp/p300-interacting transactivator 3 | *#N/A* | 234.29 | 556.00 | -1.25 | 2.20E-02 |
| Microtubule-associated protein RP/EB family member 1 | *#N/A* | 64.72 | 141.55 | -1.13 | 1.69E-02 |
| sushi domain-containing protein 2-like | *#N/A* | 393.14 | 850.71 | -1.11 | 3.95E-02 |
| Maternal B9.15 protein | *#N/A* | 66.60 | 135.40 | -1.02 | 2.89E-02 |
| UPF0676 protein C1494.01 | *#N/A* | 1063.51 | 2015.89 | -0.92 | 9.63E-03 |
| Putative ATP-dependent RNA helicase an3 | *#N/A* | 641.11 | 1095.09 | -0.77 | 4.57E-02 |
| RNA-binding protein PN | *#N/A* | 2183.61 | 1268.33 | 0.78 | 3.92E-02 |
| Pseudouridine-metabolizing bifunctional protein C1861.05 | *#N/A* | 702.90 | 406.12 | 0.79 | 4.01E-02 |
| inter-alpha-trypsin inhibitor heavy chain H3-like | *#N/A* | 42009.03 | 24189.67 | 0.80 | 3.09E-02 |
| H-2 class I histocompatibility antigen, L-D alpha chain | *#N/A* | 1368.46 | 779.88 | 0.81 | 4.13E-02 |
| Cytochrome P450 2G1 | *#N/A* | 11176.19 | 6193.01 | 0.85 | 1.39E-02 |
| Y-box-binding protein 2-B | *#N/A* | 495.59 | 269.70 | 0.88 | 2.00E-02 |
| Crystallin J1C | *#N/A* | 952.33 | 464.32 | 1.04 | 3.04E-03 |
| Nuclear ubiquitous casein and cyclin-dependent kinase substrate 1 | *#N/A* | 167.55 | 79.99 | 1.07 | 1.82E-02 |
| Cytochrome P450 3A40 | *#N/A* | 24834.84 | 10697.30 | 1.22 | 1.57E-03 |
| Reticulon-4-interacting protein 1 homolog, mitochondrial | *#N/A* | 80.06 | 32.97 | 1.28 | 1.67E-02 |
| Cytochrome P450 3A40 | *#N/A* | 2657.40 | 1047.15 | 1.34 | 5.76E-04 |
| Suppressor of hairless protein homolog | *#N/A* | 48.26 | 18.29 | 1.40 | 4.19E-02 |
| Low density lipoprotein receptor adapter protein 1-B | *#N/A* | 1698.52 | 641.91 | 1.40 | 7.65E-06 |
| saxitoxin and tetrodotoxin-binding protein 2-like | *#N/A* | 4791.57 | 1795.55 | 1.42 | 3.98E-04 |
| Serum response factor (Fragment) | *#N/A* | 120.54 | 36.77 | 1.71 | 2.56E-03 |
| Spindlin-Z | *#N/A* | 55.38 | 16.81 | 1.72 | 5.37E-03 |
| bloodthirsty-2 | *#N/A* | 29.92 | 9.02 | 1.73 | 4.57E-02 |
| Crystallin J1C | *#N/A* | 395.33 | 109.22 | 1.86 | 1.21E-02 |
| LisH domain-containing protein F | *#N/A* | 96.00 | 26.18 | 1.87 | 2.31E-04 |
| CSGA_MYXXA C-factor | *#N/A* | 41.49 | 9.25 | 2.17 | 8.87E-04 |
| Serine/threonine-protein phosphatase 4 regulatory subunit 4 | *#N/A* | 84.99 | 10.65 | 3.00 | 7.48E-05 |
| Microtubule-associated serine/threonine-protein kinase 1 | *#N/A* | 97.27 | 12.01 | 3.02 | 1.17E-05 |
| Hemoglobin subunit beta-A | *#N/A* | 7684.52 | 201.43 | 5.25 | 1.82E-02 |
| Hemoglobin subunit beta-A | *#N/A* | 18536.92 | 458.94 | 5.34 | 1.51E-02 |
| Hemoglobin subunit alpha-B | *#N/A* | 5611.05 | 125.34 | 5.48 | 1.86E-02 |
